# Lipotoxic hepatocyte derived LIMA1 enriched small extracellular vesicles promote hepatic stellate cells activation via inhibiting mitophagy

**DOI:** 10.1186/s11658-024-00596-4

**Published:** 2024-05-31

**Authors:** Shihui Li, Fuji Yang, Fang Cheng, Ling Zhu, Yongmin Yan

**Affiliations:** 1 Department of Laboratory Medicine, Wujin Hospital Affiliated With Jiangsu University, Changzhou, 213017 China; 2 Department of Laboratory Medicine, School of Medicine, Jiangsu University, Zhenjiang, 212013 China; 3 Changzhou Key Laboratory of Molecular Diagnostics and Precision Cancer Medicine, Wujin Hospital Affiliated With Jiangsu University (Wujin Clinical College of Xuzhou Medical University), Changzhou, 213017 China; 4 Wujin Institute of Molecular Diagnostics and Precision Cancer Medicine, Jiangsu University, Changzhou, 213017 China

**Keywords:** Nonalcoholic fatty liver disease, LIMA1, Hepatic stellate cells, Mitophagy, Small extracellular vesicles

## Abstract

**Background:**

Hepatic stellate cells (HSCs) play a crucial role in the development of fibrosis in non-alcoholic fatty liver disease (NAFLD). Small extracellular vesicles (sEV) act as mediators for intercellular information transfer, delivering various fibrotic factors that impact the function of HSCs in liver fibrosis. In this study, we investigated the role of lipotoxic hepatocyte derived sEV (LTH-sEV) in HSCs activation and its intrinsic mechanisms.

**Methods:**

High-fat diet (HFD) mice model was constructed to confirm the expression of LIMA1. The relationship between LIMA1-enriched LTH-sEV and LX2 activation was evaluated by measurement of fibrotic markers and related genes. Levels of mitophagy were detected using mt-keima lentivirus. The interaction between LIMA1 and PINK1 was discovered through database prediction and molecular docking. Finally, sEV was injected to investigate whether LIMA1 can accelerate HFD induced liver fibrosis in mice.

**Results:**

LIMA1 expression was upregulated in lipotoxic hepatocytes and was found to be positively associated with the expression of the HSCs activation marker α-SMA. Lipotoxicity induced by OPA led to an increase in both the level of LIMA1 protein in LTH-sEV and the release of LTH-sEV. When HSCs were treated with LTH-sEV, LIMA1 was observed to hinder LX2 mitophagy while facilitating LX2 activation. Further investigation revealed that LIMA1 derived from LTH-sEV may inhibit PINK1-Parkin-mediated mitophagy, consequently promoting HSCs activation. Knocking down LIMA1 significantly attenuates the inhibitory effects of LTH-sEV on mitophagy and the promotion of HSCs activation.

**Conclusions:**

Lipotoxic hepatocyte-derived LIMA1-enriched sEVs play a crucial role in promoting HSCs activation in NAFLD-related liver fibrosis by negatively regulating PINK1 mediated mitophagy. These findings provide new insights into the pathological mechanisms involved in the development of fibrosis in NAFLD.

**Graphical Abstract:**

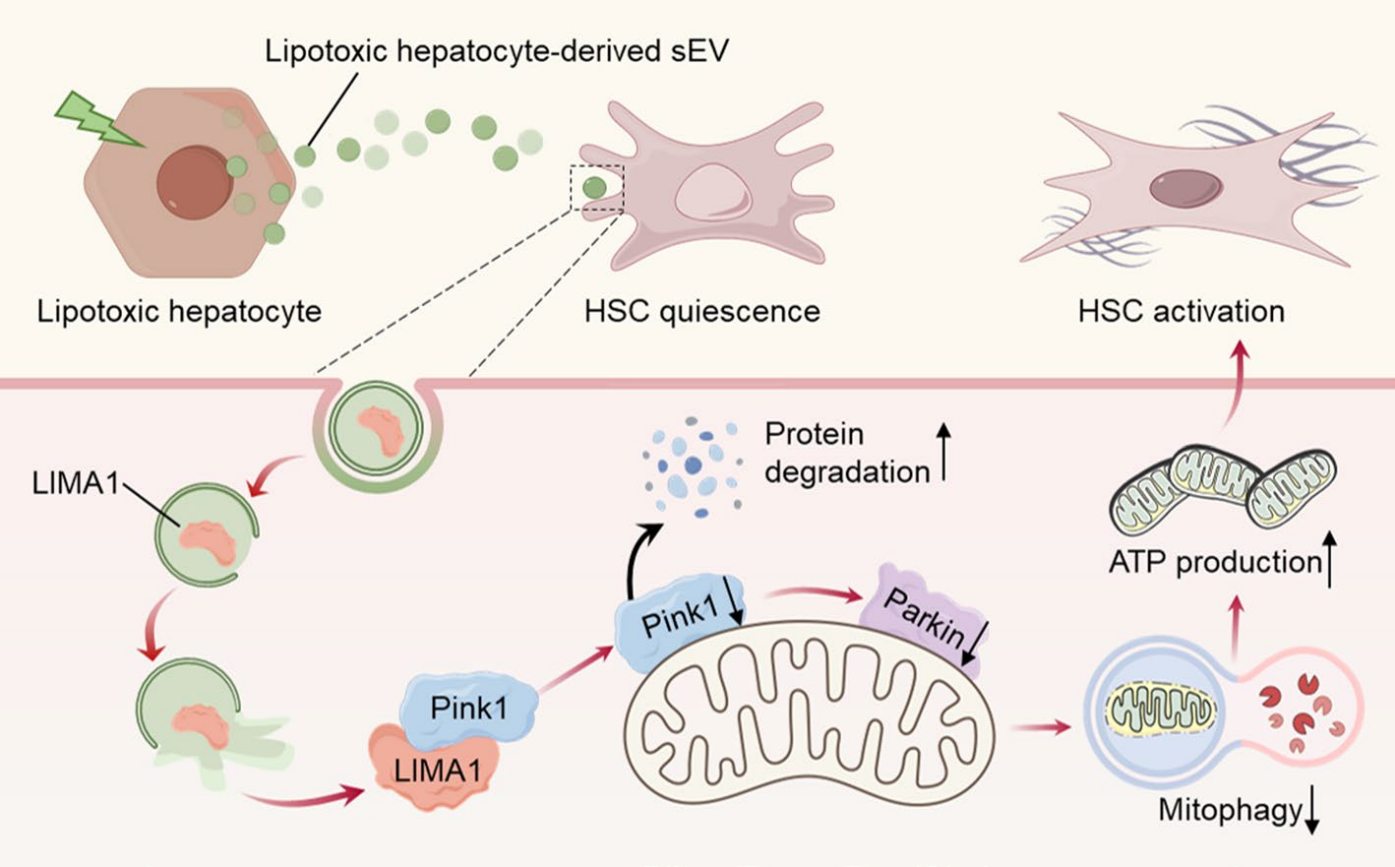

**Supplementary Information:**

The online version contains supplementary material available at 10.1186/s11658-024-00596-4.

## Background

Nonalcoholic fatty liver disease (NAFLD) has a global prevalence of approximately 25–30% [1]. NAFLD patients with advanced fibrosis are at increased risk of all-cause mortality [2]. New therapeutic treatments are required clinically because there is presently no approved medication for liver cirrhosis [3]. Activated Hepatic stellate cells (HSCs) develop into myofibroblast-like cells, which may aggravate the development of liver fibrosis. Therefore, it is vital to find out the potential molecular mechanism of HSCs activation during NAFLD development.

Small extracellular vesicles (sEV) are tiny membrane vesicles, approximately 30–150 nm in diameter, released by various cells under normal or diseased conditions. Bioactive substances that sEV transport in their cargo may have an impact on recipient cells’ metabolisms [4]. Massive sEV enriched in protein and miRNA that were released from lipotoxic hepatocyte were then absorbed by HSCs, ultimately resulting in the progression of hepatic fibrosis [5, 6]. It is crucial to explore the key fibrotic factors that shuttled from sEV to HSCs for understanding NAFLD-related fibrosis [7–10]. Growing empirical evidence suggests that dysfunction in the mitochondria of HSCs can trigger the activation of HSCs, thereby leading to the development of liver fibrosis. Administration of resveratrol has been observed to induce mitophagy in HSCs, presenting a potential therapeutic avenue for liver fibrosis [11]. Activation of mitophagy is related to the regression of liver fibrosis through the initiation of apoptosis in HSCs [12]. However, the molecular mechanisms of lipotoxic hepatocyte derived sEV govern mitophagy in HSCs and liver fibrosis remain poorly understood.

LIMA1 (LIM Domain And Actin Binding 1), also known as EPLIN, is an actin-binding cytoskeletal protein containing a LIM structural domain [13]. Currently, studies on LIMA1 mainly focus on cancer progression and intestinal cholesterol absorption [14, 15], whereas the role of LIMA1 in HSCs activation and liver fibrosis remain virtually unknown. In this study, we investigated how LTH-sEV delivered LIMA1 affects the activation of HSCs. Our findings suggest that OPA-induced lipotoxicity led to higher levels of LIMA1 protein in LTH-sEV and an increased release of these vesicles. Additionally, we propose that LIMA1 may have a significant role in promoting HSC activation by potentially inhibiting PINK1-Parkin-mediated mitophagy. This research offers new insights into understanding the progression of liver fibrosis in NAFLD.

## Materials and methods

### Cell culture

Human hepatic stellate cell line LX2 (#CC4023) was purchased from Saiku Culture Bank (Guangzhou, China) and cultured in H-DMEM (Cytiva, USA, AI30093259) with 10% FBS (ExCell, USA, FND50). Human hepatic cell line L02 (#CL0192) was purchased from Fenghui Biology Co., Ltd (Changsha, China) and cultured in RPMI 1640 (Gibco, USA, C11875500) containing 10% FBS. All cells were grown in the incubator at 37 °C with saturated humidity and 5% CO2.

### Isolation and cultures of primary mouse hepatocyte

Primary mouse hepatocytes were isolated from the liver of 6-week-old C57BL/6 male mice. For hepatocyte isolation, primary mouse hepatocytes were isolated using a standard two-step collagenase perfusion method [16]. Mice were anesthetized by intraperitoneal injection of sodium pentobarbital (100 mg/kg). A catheter was then inserted into the exposed inferior vena cava and the portal vein drainage was incised. The liver was perfused with Hank’s Balanced Salt Solution (HBSS)-EGTA solution (BOSTER, Wuhan, China, PYG0079) for 10 min, followed by infusion with 100 U/mL collagenase IV (Biofroxx, Einhausen, Germany, 2275GR001). After 15 min of perfusion, the livers were collected and minced to release hepatocytes. The resulting cell suspension was filtered through a sterile 100 μm cell strainer Falcon (Corning, USA, 352,360) and centrifuged at 150 ×*g* for 5 min. These cells were cultured in H-DMEM medium (Cytiva, USA, AI30093259) containing 10% FBS (ExCell, USA, FND50), supplemented with 1 × penicillin–streptomycin solution (Biosharp, China, BL505A) at 37 °C in a humidified incubator containing 5% CO_2_.

### High-fat Diet (HFD) induced mice model

Male C57BL/6 mice were maintained in a pathogen-free environment with a temperature of 21–23 °C, relative humidity of 50–60%, and a 12 h light and dark cycle. The mice model of HFD-induced NAFLD was generated as described in previous study [17]. The mice were randomly assigned to four groups (6 mice per group). The groups were as follows: the NCD group, which was fed a normal chow diet; the HFD 4w group, which was fed a high-fat diet for 4 weeks; the HFD 8w group, which was fed a high-fat diet for 8 weeks; and the HFD 12w group, which was fed a high-fat diet for 12 weeks. The normal chow feed, which contained 10% kcal from fat, and the HFD feed, which contained 45% kcal from fat, were purchased from Medicience (Jiangsu, China).

### Fatty acid treatment

Combination of palmitic acid (Sigma, USA, P0500) and oleic acid (Sigma, USA, O7501) was performed to induce lipotoxic hepatocyte according to the standard protocol described previously [18]. Briefly, after reached 80% confluence, 660 μM oleic acid conjugated with BSA and 330 μM palmitic acid conjugated with BSA was added to the cultured medium for treating L02 cells for 8 h, 16 h, 24 h to develop lipotoxic L02 cells.

### Immunohistochemistry

Following dewaxing and rehydration of the paraffin sections, antigen retrieval was performed using citric acid buffer (10 mM citric acid, 0.05% Tween 20, pH = 6.0). The endogenous peroxidase activity was inactivated by treating with 3% H_2_O_2_ for 30 min. To prevent non-specific binding of antibodies, a blocking solution of 5% BSA was applied to the slides. The liver tissue sections were then incubated overnight at 4 °C with primary antibodies against LIMA1 (1:100, Proteintech) and α-SMA (1:100, Proteintech). Subsequently, the sections were incubated with biotin-conjugated anti-rabbit IgG and streptavidin–biotin. Finally, the liver tissue sections were visualized using the DAB Horseradish Peroxidase Color Development Kit (BOSTER, Wuhan, China, 18E16B22) and counterstained with hematoxylin. Random fields were captured using a pathological section scanner (InteMedic, Guangzhou, China).

### Western blot

RIPA buffer (Beyotime, Shanghai, China, P0013K) containing 1% protease inhibitor PMSF (Biosharp, Anhui, China, BL507A-S) was used to lyse samples from different groups at 4℃. After centrifugation, the supernatants were collected. The lysates were brought to a boil while adding loading buffer (0.25 M Tris–HCl pH 6.8, 1 M sucrose, 5 mM EDTA, 0.1% bromophenol blue, 10% SDS, and 5% β-mercaptoethanol). Protein (30 μg) was added in equal proportions to 8–12% Bis–Tris gels and then transferred to polyvinylidene difluoride membranes. After transfer, the membranes were treated with blocking buffer (PBS, 0.1% Tween20, and 5% BSA) for 2 h. The primary and secondary antibodies used in this study are listed in Additional file 2: Table S1. Protein bands were visualized using an enhanced chemiluminescence (ECL) solution (Beyotime, Shanghai, China, P0018M) according to the manufacturer’s instructions and quantified using Image J software.

### Quantitative RT-PCR (qRT-PCR)

The total RNA of LX2 cells was extracted using Trizol (GIBCO, USA, 15,596–026) following the manufacturer’s instructions. For the reverse transcription of RNA into cDNA, 1 µg of total RNA was used in a reaction with the SuperScript Reverse Transcriptase Kit (Vazyme, Nanjing, China, Q511-02) according to the manufacturer’s instructions. qRT-PCR assays were performed using SYBR Green (CWBIO, Beijing, China, CW0659). The sequences of the qPCR primers used in this study can be found in Additional file 2: Table S2. The fluorescence signals were detected using the CFX96 Touch™ Real-Time PCR Detection System (Bio-Rad, USA). All measurements were normalized using β-actin expression as an internal standard, and relative expression was calculated using the 2^−ΔΔCt^ method.

### Oil Red O staining

To detect lipid droplets, cells were stained with Oil Red O (Sigma, USA, O0625). A 0.5% Oil Red O Stock Solution (in isopropanol) was used. The staining solution consisted of 6 parts of stock Oil Red O and 4 parts of distilled water, which was filtered with filter paper before use. After fixing the cells in PFA, they were stained with 1 ml of the staining solution for 20 min. The background was then cleared using 60% isopropanol. Nuclei were stained with hematoxylin and the samples were sealed for observation.

### Nile Red staining

The Nile red staining assay utilized a stock solution consisting of 1 mg of Nile red (Sigma, USA, 72,485) dissolved in 1 ml of 100% acetone. The OPA treated L02 cells were then stained with a 1 ml working solution of fluorescence Nile red (diluted at 1:5000) for 30 min at room temperature. The stained cells were subsequently analyzed using a fluorescence microscope (Olympus, Tokyo, Japan).

### FDA fluorescence

The cells were removed from the culture medium and washed three times with PBS. The cells were then treated with 10 μg/mL FDA (BOSTER, Wuhan, China, AR1186), placed in a dark place at room temperature for 5 min for staining, and imaged by fluorescence microscope (Olympus, Tokyo, Japan).

### Lentiviral knockdown of LIMA1 in L02 cells

L02 cells were infected with a Lentiviral vector (pLKO.2-U6-L02-hPGK-copGFP-Puro-shLIMA1) containing LIMA1 shRNA sequence (sequence: GTC TCT GAA TTG GTC GAG TTT) or a negative control vector (pLKO.1-Puro-shRNA) purchased from Vigen Biotech (Zhenjiang, China). The infection was done at a multiplicity of infection (MOI) of 20% and 50% confluency. The expression of green fluorescent protein (GFP) was observed using fluorescence microscopy. To remove untransfected cells, puromycin was added at a final concentration of 1 µg/ml within 96 h after infection.

### Isolation and identification of sEV

The sEV were isolated from cell culture medium by differential centrifugation. Briefly, L02 cells and LIMA1 stable knockdown L02 cells were cultured in 100 mm dishes, treated with 660 μM oleic acid and 330 μM palmitic acid, and then replaced with FBS-free medium to continue culturing for 24 h. The conditioned medium was collected and centrifuged at 300*g* for 10 min, at 2000*g* for 10 min, and at 10,000*g* for 30 min to remove cells and cell debris. Supernatants were then concentrated using a 100 kDa molecular weight cut-off (MWCO) ultrafiltration filter according to the manufacturer’s instructions (Millipore, USA). The concentrated supernatant was ultracentrifuged at 100,000*g* for 70 min (Optima L-90 K; Beckman Coulter, Brea, CA, USA). The sEV-enriched pellet was collected from the bottom of the tube and resuspended in PBS, and the final sEV were passed through a 0.22 μm filter and stored at -80 °C. The obtained sEV were named LTH-sEV and LTH-sEV^shLIMA1^ respectively. The supernatant of normally cultured L02 cells was collected and the extracted sEV were named L02-sEV. In addition, the supernatant of OPA-treated primary mouse hepatocytes was collected, and the extracted sEV were named pLTH-sEV. The protein concentration of the extracted sEV was quantified by a BCA protein assay kit (Pierce, ThermoFisher). The morphology of sEV was observed by transmission electron microscopy (TEM). The amount and size distribution of sEV was measured by NanoSight tracking analysis (NTA) with NTA 3.1 Software (NanoSight, Malvern, UK). Expression of sEV markers TSG101, CD63, and CD9, and endoplasmic reticulum protein Calnexin was analyzed by western blot.

### Co-culture of sEV with LX2

LX2 were cultured in 6-well plates until it reached approximately 50–60% confluence. LTH-sEV were then co-cultured with LX2 at different concentrations and divided into 4 groups: control group, LTH-sEV 50 μg/ml group, LTH-sEV 100 μg/ml group and LTH-sEV 200 μg/ml group. Similarly, L02-sEV were co-cultured with LX2 at different concentrations and divided into 4 groups: control group, L02-sEV 50 μg/ml group, L02-sEV 100 μg/ml group and L02-sEV 200 μg/ml group. These cells were treated for 48 h for further investigation. In addition, LX2 was co-cultured with pLTH-sEV (200 μg/ml) for 48 h. To verify the role of mitochondrial autophagy in LTH-sEV-treated LX2, the ctrl group, the LTH-sEV group, and the LTH-sEV (200 μg/ml) + Urolithin A (50 μM) group were set up.

### Immunofluorescence

The cells were fixed with 4% paraformaldehyde for 20 min, followed by permeabilization in PBS containing 0.1% Triton X-100 (Beyotime, Shanghai, China, P0096) for 10 min. After being washed twice with PBS, the cells were incubated with a 5% BSA solution for 30 min to block nonspecific binding sites. Liver tissue sections and cultured cells were then incubated with specific primary antibodies. Subsequently, the slides were incubated with the secondary antibody for 30 min at room temperature and in the dark. The primary and secondary antibodies used for immunofluorescence are provided in Additional file 2: Table S1. The nuclei were stained with Hoechst 33,342, and fluorescence images were acquired using a fluorescence microscope (Olympus, Tokyo, Japan) in a random field of view.

### Cell counting kit-8 (CCK-8) assay

Cell proliferation was assessed using the CCK-8 assay (Beyotime, Shanghai, China, BS350A) following the standard procedure recommended by the reagent manufacturer. LX2 were seeded onto 96-well plates at a density of 2 × 10^3^ cells per well and subjected to different treatments. After incubation at 37 °C for approximately 2 h, 10 μl CCK-8 solution was added to each well. The absorbance values at 450 nm were measured using a multi-function microplate reader (ThermoScientific, Waltham, MA, USA).

### sEV internalization assay

PKH26 dye (Solarbio, Beijing, China, D0030) was used to label sEV according to the manufacturer’s instructions. LX2 were incubated with PKH26-labelled sEV for 6 h, and then fixed with 4% paraformaldehyde. The actin cytoskeleton was visualized using phalloidin (Solarbio, Beijing, China, CA1610), and nuclei were detected using Hoechst 33,342 dye. Fluorescence microscopy (Olympus, Tokyo, Japan) was employed to evaluate the internalization of sEV.

### Cell transfection of plasmid and siRNA

For overexpression of LIMA1, the LIMA1 expression vector (LIMA1-pcDNA3.1-3xFlag-C) and control vector were purchased from Fenghui Biology Co., Ltd. (Changsha, China) and transfected into LX2. For LIMA1-knockdown cell lines, control scrambled siRNA and Homo-LIMA1 siRNA (siLIMA1) sequences (Additional file 2: Table S3) were purchased from GenePharma Co., Ltd. (Shanghai, China). Cells were transiently transfected by use of Lipofectamine 2000 reagent (Invitrogen, USA, 11,668,500) according to the manufacturer’s instructions. At 48 h after transfection, cells were harvested and used for experiments.

### Construction of mt-Keima-COX8 transfected LX2

For the detection of mitophagy, mt-Keima-COX8 lentivirus titration solution samples were purchased from Vigen Biotech (Zhenjiang, China). The cell fusion rate was about 70% when the virus was infected. Lentivirus stocks were carefully diluted in FBS-free culture medium. Add the calculated virus solution to LX2, mix well and place in a carbon dioxide incubator (37 °C, 5% CO_2_). Selecting infected cells with puromycin at an appropriate concentration after 48 h.

### Transmission electronic microscopy

The cells were processed and collected following the provided instructions, and subsequently fixed in 2.5% glutaraldehyde at 4 °C for an overnight duration. Following this, the cells underwent three washes with PBS and were then fixed in 2% osmium tetroxide for a period of 1.5 h at room temperature. The cells were then subjected to dehydration through a series of ethanol concentrations, followed by embedding and fixation, and ultimately stained with uranyl acetate/lead citrate. Finally, the sections were examined using a transmission electron microscope.

### ATP assay

To determine the ATP content, an ATP colorimetric assay kit (Beyotime, Shanghai, China, S0027) was used following the manufacturer’s instructions. LX2 were lysed with lysate after being rinsed with PBS, and then centrifuged at 12,000*g* for 5 min. All reactions were carried out in 96-well plates by mixing 20 μl of the lysis supernatant with 100 μl of the ATP assay working solution. The Luminance (RLU) was measured immediately using a fluorescent chemical analyzer (ThermoScientific, Waltham, MA, USA).

### Measurement of mitochondrial membrane potential (ΔΨm)

Mitochondrial membrane potential (ΔΨm) was assessed using the JC-1 fluorescence probe (Beyotime, Shanghai, China, C2003S). The JC-1 probe was diluted in fresh medium at a ratio of 1:1000 and incubated with cells in the dark at 37 °C for 20 min. After fixation with 4% paraformaldehyde, LX2 were stained with Hoechst 33,342. Fluorescence images were captured using a fluorescence microscope (Olympus, Tokyo, Japan) in a randomly selected field of view.

### Mitochondrial autophagy promoters and mitochondrial autophagy inhibitors

To investigate the role of mitophagy in HSCs activation, we conducted experiments using mitophagy inhibitors Liensinine (Solarbio, Beijing, China, IL0640, 50 μM) and mitophagy promoters Urolithin A (Solarbio, Beijing, China, IU0200, 50 μM) in co-cultivation with LX2 for 48 h. Subsequently, protein and RNA were collected for further analysis.

### Protein–protein docking

The X-ray crystal structures of LIMA1 were retrieved from the Protein Data Bank. The predicted structures of PINK1 were generated by Alphafold. To ensure the accuracy of the docking results, the protein was prepared by the AutoDockTools-1.5.7, and the water molecules were manually eliminated from the protein and the polar hydrogen was added. Docking Web Server (GRAMM) was used for protein–protein docking. The resulting protein–protein complex was also manually optimized by removing water and adding polar hydrogen by the AutoDockTools-1.5.7. Finally, the protein–protein interactions were predicted and the protein–protein interaction figure was generated by PyMOL (https://pymol.org).

### Co-immunoprecipitation (Co-IP))

Samples for Co-IP were suspended in lysis buffer (50 mM Tris–HCl, pH 7.4, 0.5% NP-40, 150 mM NaCl, 1 mM EDTA, 10% glycerophosphate) at 4 °C for 30 min. Cell lysates were incubated with the anti-LIMA1 antibodies for 2 h. Then protein A/G agarose beads (Beyotime, Shanghai, China, P2197S) were added and the mixtures allowed incubating at 4 °C with gentle rocking overnight. Beads were washed with an appropriate amount of lysis buffer and boiled with 2 × SDS loading buffer (0.25 M Tris–HCl pH 6.8, 1 M sucrose, 5 mM EDTA, 0.1% bromophenol blue, 10% SDS, and 5% β-mercaptoethanol) before analysis by Western blot.

### Cycloheximide chase assay

The half-life of PINK1 protein in LX2 was determined using a cycloheximide (CHX)-chase assay. This assay allows for the examination of protein degradation over a specific time period. LX2 were seeded in six-well cell culture plates and transfected with LIMA1 overexpression plasmid and LIMA1 small interfering RNA. After 48 h of transfection, the cells were treated with 20 μg/ml CHX (Sigma-Aldrich, Shanghai, China, C104450) for different time-points. Subsequently, the cells were collected and analyzed using Western blot.

### sEV injection in HFD mice

For the sEV-injected mice model, mice were randomly divided into four groups (6 mice per group): NCD group, mice fed with normal chow diet for 14 weeks; HFD group, mice fed with high-fat diet for 14 weeks; HFD + LTH-sEV^shCtr^ group, HFD mice injected with LTH-sEV^shCtr^; HFD + LTH-sEV^shLIMA1^ group, HFD mice injected with LTH-sEV^shLIMA1^. LTH-sEV^shCtr^ (10 mg/kg) and LTH-sEV^shLIMA1^ (10 mg/kg) were injected once a week for four consecutive weeks through the tail vein in HFD-fed mice starting at 10 weeks. All mice were euthanized to collect blood and liver samples for further analysis.

### Masson staining and Sirius Red staining

The collected specimens were fixed in a 10% formaldehyde solution for 24 h. Sections of 4 μm thickness were prepared after paraffin embedding. To examine histopathological changes in the liver, sections from each group were deparaffinized with xylene, rehydrated with varying concentrations of ethanol, and then stained with the Masson trichromatic staining kit (Solarbio, Beijing, China, G1340) and Sirius red staining kit (Solarbio, Beijing, China, G1473) following the manufacturer's instructions. The samples were observed under a pathological section scanner (InteMedic, Guangzhou, China).

### Statistical analysis

Results were presented as the mean ± SD from at least three independent experiments. Normality of data was tested with the Shapiro–Wilk test. Statistical analysis was conducted using GraphPad Prism 8.0 software (GraphPad, San Diego, CA) with Student’s t-test for comparisons between two groups or One-way analysis of variance (ANOVA) with Tukey’s post hoc test for comparisons among multiple groups. *P* value ≤ 0.05 was considered statistically significant.

## Results

### LIMA1 is upregulated in HFD-induced fatty liver and in sEV from lipotoxic hepatocyte

**Fig. 1:**
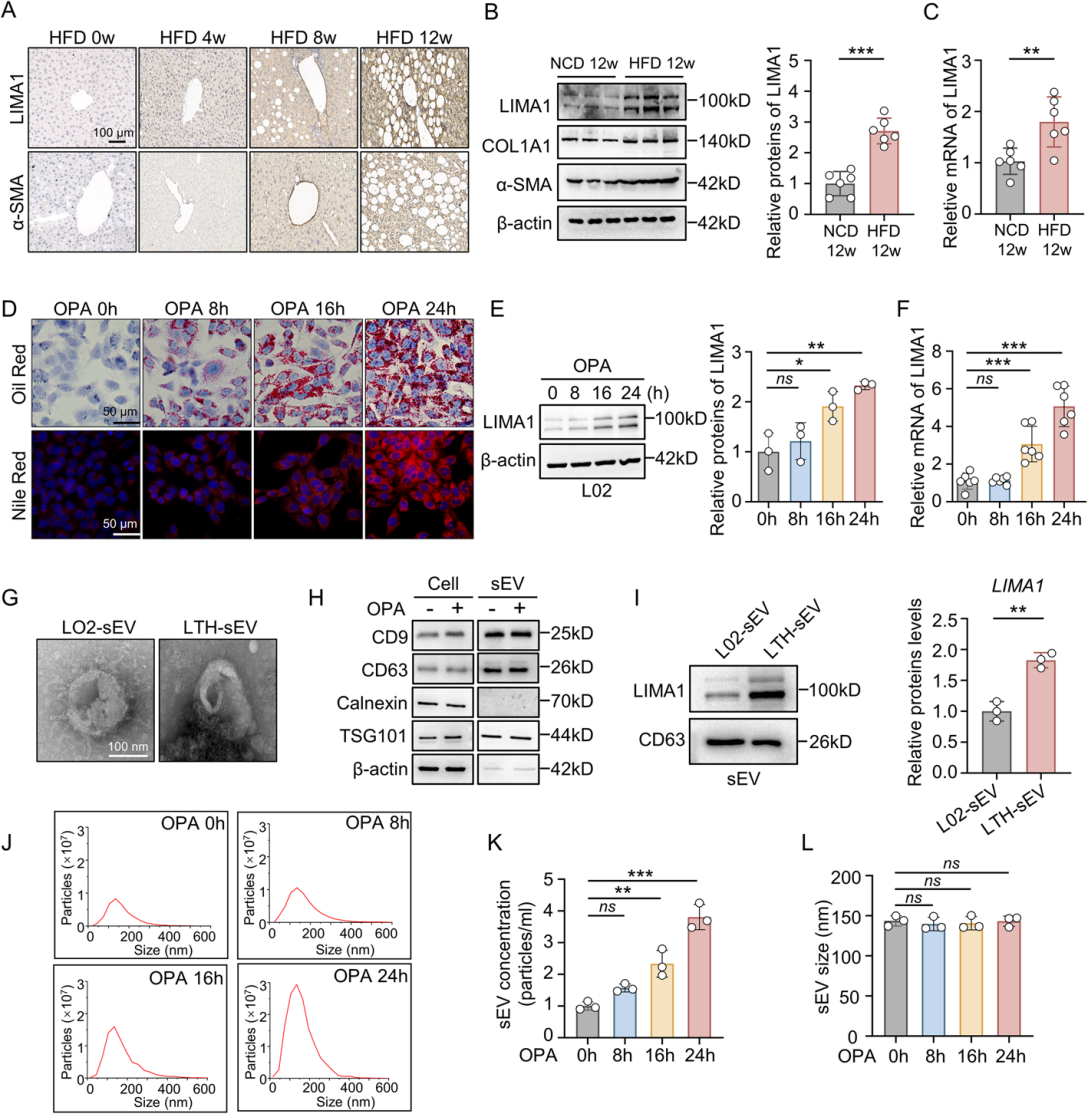
LIMA1 is upregulated in lipotoxic hepatocyte and lipotoxic hepatocyte-derived sEV. **A** Immunohistochemistry of LIMA1 and α-SMA in livers from HFD mice at 0, 4, 8, and 12 weeks. Scale bar = 100 μm, (*n* = 6 mice per group). **B** Western blot of LIMA1, α-SMA and COL1A1 in livers from HFD mice, (*n* = 6 mice per group). **C** qRT-PCR of LIMA1 mRNA in livers from NCD and HFD mice at 12 weeks, (*n* = 6 mice per group). **D** Oil Red O and Nile Red staining of intracellular lipid droplets in OPA treated L02 cells at 0, 8, 16, and 24 h. Scale bar = 50 μm. **E** Western blot of LIMA1 in OPA treated cells. **F** qRT-PCR of LIMA1 mRNA expression in OPA treated L02 cells. **G** TEM of sEV isolated from normal L02 cells (L02-sEV) and OPA-treated L02 cells (LTH-sEV). Scale bar = 100 nm. **H** Western blot of sEV markers CD9, CD63, TSG101 and endoplasmic reticulum marker Calnexin in LTH-sEV. **I** Western blot of LIMA1 and CD63 in LTH-sEV and L02-sEV. **J** The size and concentration of LTH-sEV were determined by nanoparticle tracking analysis. **K** The concentration of LTH-sEV after OPA treatment of L02 for different times. **L** Size distribution of LTH-sEV after OPA treatment of L02 for different times. All data were expressed as the means ± SD of at least 3 independent experiments, *ns*: no significance; *P < 0.05; **P < 0.01; ***P < 0.001

NAFLD transcriptomic data (GSE135251) was used to investigate the molecular mechanism underlying NAFLD progression induced by 206 NAFLD cases with different fibrosis stages and 10 controls. Analysis of differentially expressed genes in frozen biopsies revealed a significant increase in LIMA1 mRNA expression (Additional file 1: Fig. S1). The association between LIMA1 expression in lipotoxic hepatocyte and hepatic stellate cell activation was observed. Immunohistochemistry results showed sequential increase in the expression of LIMA1 in mice hepatocyte and α-SMA in myofibroblasts after 4, 8, and 12 weeks of HFD induction (Fig. 1A). qRT-PCR and Western blot analysis also demonstrated a significant increase in LIMA1 mRNA and protein expression in the liver tissue of HFD-fed mice, along with increased protein expression of fibrosis-related proteins COL1A1 and α-SMA (Fig. 1B, C). Immunofluorescence staining showed that the cell types overexpressing LIMA1 in the liver of HFD mice were mainly albumin-positive hepatocytes and α-SMA-positive hepatic stellate cells, with a small amount present in Kupffer cells (Additional file 1: Figure S2). In OPA-induced lipotoxic damaged hepatocyte, there was an increase in lipid deposition (Fig. 1D) and hepatocytes death occurred 24 h after OPA treatment (Additional file 1: Fig. S3A–C). Notably, LIMA1 mRNA and protein expression increased significantly with OPA induction (Fig. 1E, F and Additional file 1: Fig. S4A, B). sEV, an important carrier of intercellular communication, plays a crucial role in hepatic stellate cell activation and liver fibrosis progression. sEV isolated from normal L02 cells and lipotoxicly damaged L02 cells (LTH-sEV) were compared for their LIMA1 expression levels. TEM and Western blot analysis confirmed that L02-sEV and LTH-sEV were tiny membrane vesicles around 100 nm in diameter, expressing positive markers CD9 and CD63 proteins of sEV, while not expressing the negative marker endoplasmic reticulum Calnexin protein (Fig. 1G, H). LIMA1 expression levels were significantly higher in LTH-sEV compared to L02-sEV (Fig. 1I). Nanoparticle tracking analysis (NTA) showed that with the prolongation of OPA treatment time, the amount of LTH-sEV released from hepatocytes increased significantly (Fig. 1J, K) but the peak of the nanoparticle size distribution never altered (Fig. 1L). pLTH-sEV exhibited similar particle sizes to LTH-sEV derived from L02 cells (Additional file 1: Fig. S4C, D). These findings suggest that LIMA1 expression increases in lipotoxically injured liver tissue and positively associated with the expression of hepatic stellate cell activation marker α-SMA. Fatty acid treatment induces the release of sEV from hepatocyte and increases LIMA1 expression in sEV.

### LIMA1 enriched sEV increases LIMA1 protein expression in HSCs and induces their activation.

**Fig. 2:**
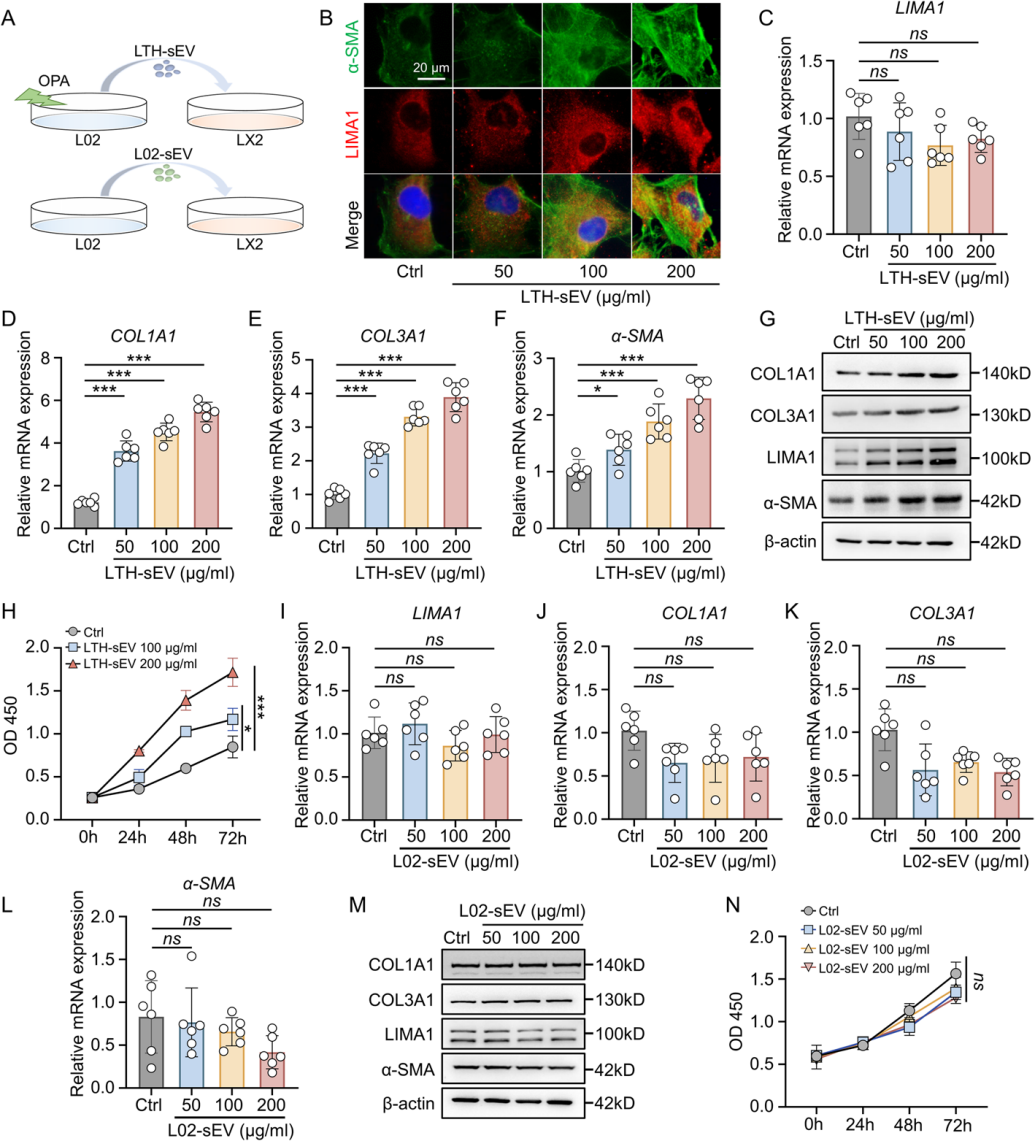
LTH-sEV induce LX2 activation and LIMA1 expression. **A** Schematic diagram of the sEV stimulation experiment in vitro. **B** Immunofluorescence staining of α-SMA (green) and LIMA1 (red) in LX2 after accepting LTH-sEV. Scale bar = 20 μm. **C-F** qRT-PCR of LIMA1, COL1A1, COL3A1 and α-SMA mRNA in LX2 treated with LTH-sEV. **G** Western blot of LIMA1, COL1A1, COL3A1 and α-SMA in LX2 treated with LTH-sEV. **H** LX2 treated with LTH-sEV was detected by CCK-8 assay as indicated. **I-L** qRT-PCR of LIMA1, COL1A1, COL3A1 and α-SMA mRNA expression in LX2 treated with L02-sEV. **M** Western blot of LIMA1, COL1A1, COL3A1 and α-SMA expression in LX2 treated with L02-sEV. **N** LX2 treated with L02-sEV was detected by CCK-8 assay as indicated. All data were expressed as the means ± SD of at least 3 independent experiments, *ns*: no significance; *P < 0.05; **P < 0.001

To investigate the regulatory impact of LTH-sEV on the activation of HSCs, we subjected a human hepatic stellate cell line (LX2) to varying concentrations of LTH-sEV or L02-sEV for a duration of 24 h (Fig. 2A). The findings from immunofluorescence analysis indicated the simultaneous presence of LIMA1 and α-SMA protein expressions in LX2 treated with LTH-sEV (Fig. 2B). We found that the concentration of LTH-sEV did not affect the level of LIMA1 mRNA in LX2 (Fig. 2C). However, the mRNA expression of hepatic stellate cell activation markers, such as COL1A1, COL3A1, and α-SMA, increased with the increasing concentration of LTH-sEV and pLTH-sEV (Fig. 2D–F and Additional file 1: Fig. S4E–G). Additionally, Western blot analysis demonstrated that the protein levels of LIMA1, COL1A1, COL3A1, and α-SMA in LX2 were elevated following treatment with varying concentrations of LTH-sEV and pLTH-sEV (Fig. 2G and Additional file 1: Fig. S4H). When the liver is stimulated by pathological factors, HSCs are activated and transformed into myofibroblasts (MFB), leading to cell proliferation and fibrosis development. Therefore, we further investigated the effect of LTH-sEV on LX2 proliferation. The results from the CCK8 assay demonstrated that LTH-sEV significantly promoted the proliferation of LX2 (Fig. 2H). Administration of OPA did not lead to the overexpression of LIMA1 (Additional file 1: Fig. S5A, B) or the release of sEV in LX2 (Additional file 1: Fig. S6A, B). To gain a deeper understanding of the role of LIMA1 in LTH-sEV-mediated HSCs activation, we also analyzed the effect of L02-sEV on LX2 activation. Interestingly, treatment with L02-sEV at the same concentration and time did not result in significant changes in the mRNA and protein levels of LIMA1, COL1A1, COL3A1, and α-SMA (Fig. 2I–M). Furthermore, L02-sEV did not have a promoting effect on LX2 proliferation (Fig. 2N). These results suggest that LIMA1 enriched LTH-sEV plays a more prominent role in promoting HSC activation compared to L02-sEV.

### LIMA1 plays a crucial role in LTH-sEV promoted HSCs activation.

**Fig. 3:**
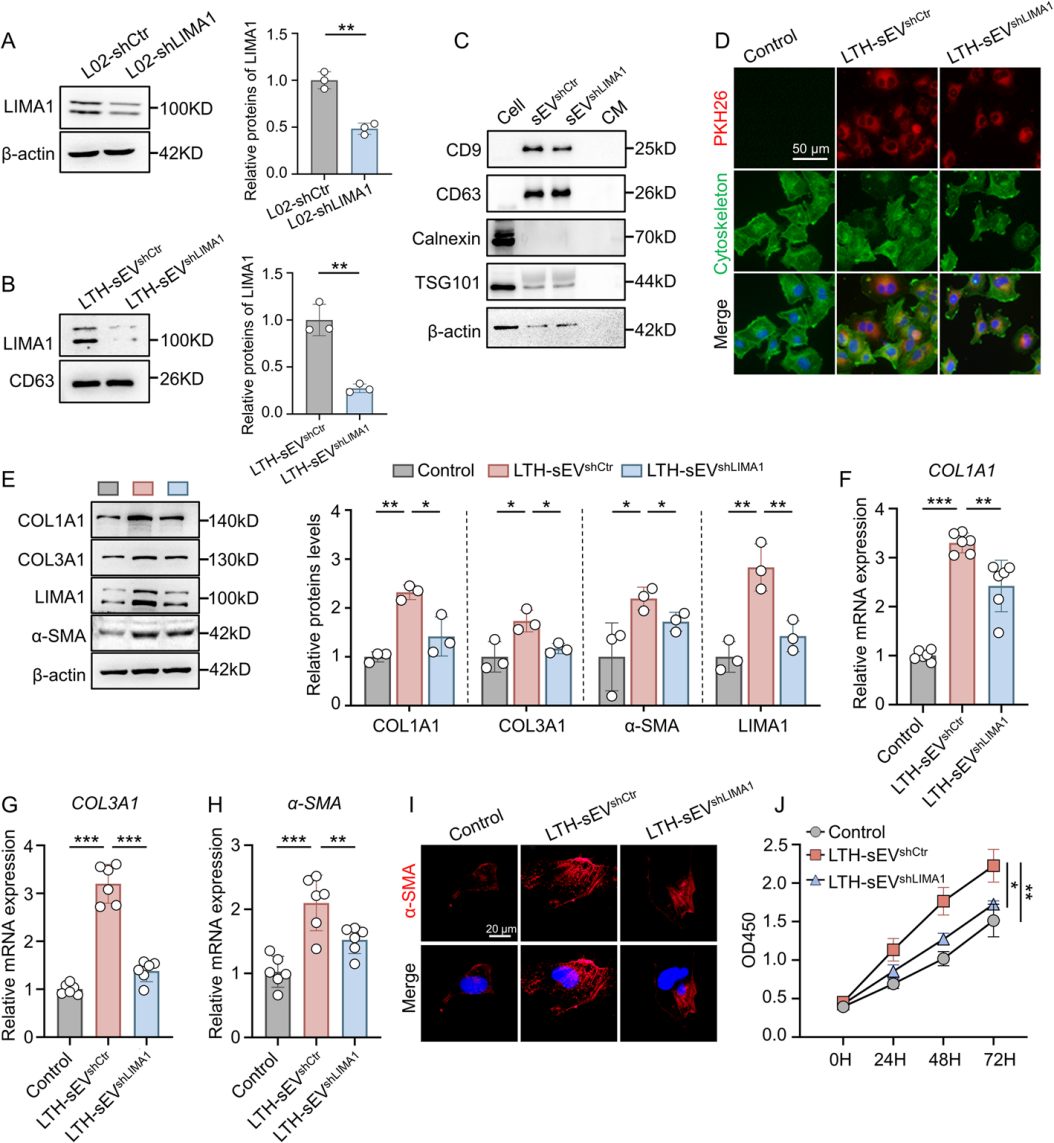
LTH-sEV activates LX2 by transferring LIMA1 to LX2. **A** Western blot of LIMA1 in L02-shCtr and L02-shLIMA1. **B** Western blot of LIMA1 in LTH-sEV^shCtr^ and LTH-sEV^shLIMA1^. **C** Western blot of sEV markers CD9, CD63, TSG101 and endoplasmic reticulum marker Calnexin in LTH-sEV^shCtr^ and LTH-sEV^shLIMA1^. **D** Internalization of LTH-sEV^shCtr^ and LTH-sEV^shLIMA1^ labeled with PKH26 into LX2. Scale bar = 50 μm. **E** Western blot of COL1A1, COL3A1, α-SMA and LIMA1 in LX2 treated with LTH-sEV^shCtr^ or LTH-sEV^shLIMA1^. **F–H** qRT-PCR of COL1A1, COL3A1 and α-SMA mRNA in LX2 treated with LTH-sEV^shCtr^ or LTH-sEV^shLIMA1^. **I** Immunofluorescence staining of α-SMA (red) in LX2 after accepting LTH-sEV^shCtr^ or LTH-sEV^shLIMA1^. Scale bar = 20 μm. **J** Cell proliferation of LX2 treated with LTH-sEV^shCtr^ or LTH-sEV^shLIMA1^ were determined by CCK-8 assays. All data were expressed as the means ± SD of at least 3 independent experiments, * *P* < 0.05; **P < 0.01; ***P < 0.001

**Fig. 4:**
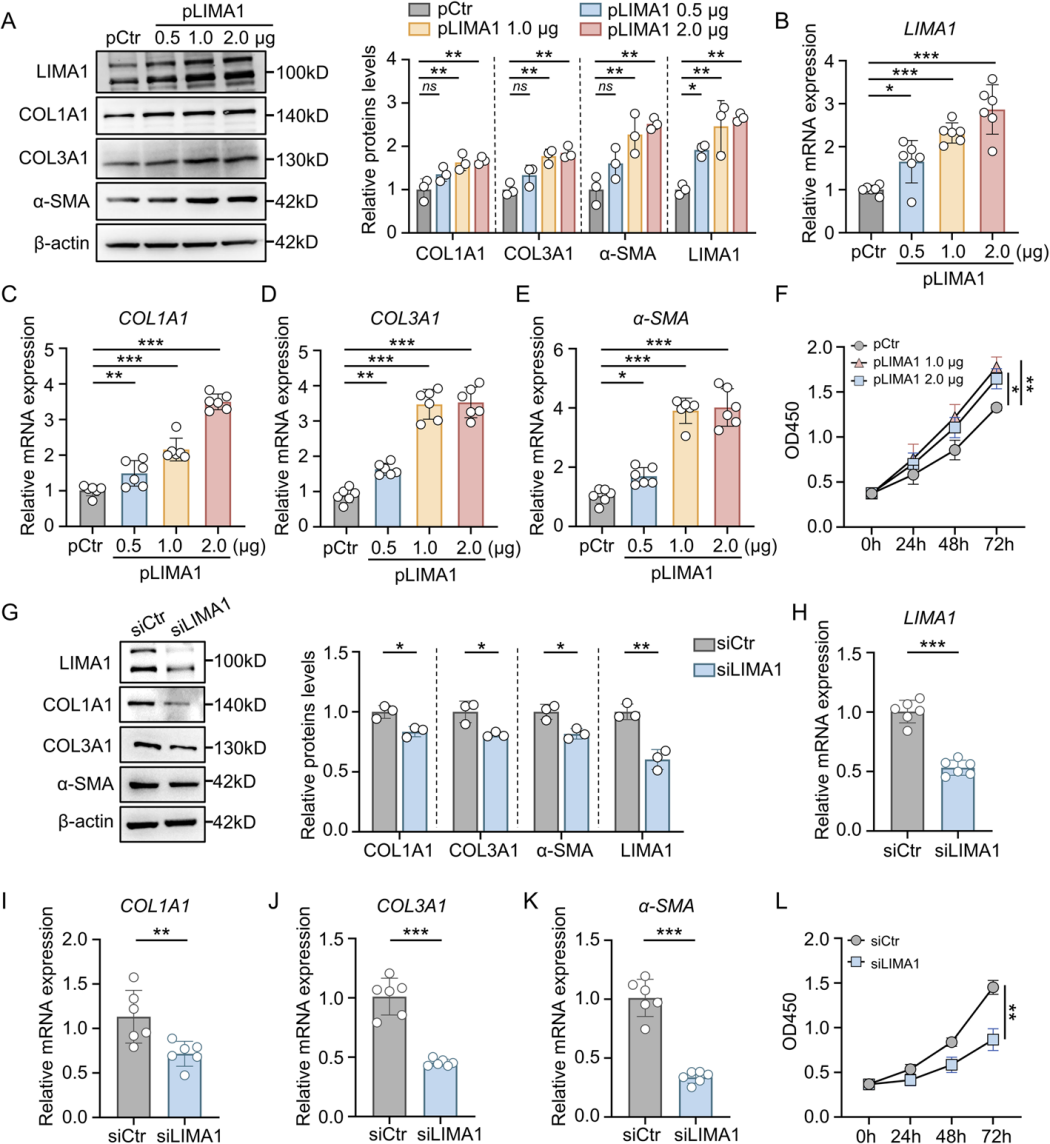
LIMA1 overexpression and knockdown promote or inhibit HSCs activation. **A** Western blot of COL1A1, COL3A1, α-SMA and LIMA1 in LIMA1-overexpressing plasmid-transfected LX2. **B**–**E** qRT-PCR of LIMA1, COL1A1, COL3A1 and α-SMA mRNA in LIMA1-overexpressing plasmid-transfected LX2. **F** Cell viability detection of LX2 overexpressing LIMA1 were determined by CCK-8 assays. **G** Western blot of COL1A1, COL3A1, α-SMA and LIMA1 in LIMA1-knockdown LX2. **H**–**K** qRT-PCR of LIMA1, COL1A1, COL3A1 and α-SMA mRNA in LIMA1-knockdown LX2. **L** Cell viability detection of LIMA1-knockdown LX2 were determined by CCK-8 assays. All data were expressed as the means ± SD of at least 3 independent experiments, *P < 0.05; **P < 0.01; ***P < 0.001

To investigate the involvement of LIMA1 in the regulation of HSCs activation by LTH-sEV, we used recombinant LIMA1-shRNA lentivirus to infect hepatocyte, constructed LIMA1 stably knockdown hepatocyte. In addition, the efficiency of lentiviral transfection was also verified (Fig. 3A). sEV (referred to as LTH-sEV^shLIMA1^) were isolated from LIMA1 knockdown hepatocyte. The verification of sEV characteristic proteins and the efficiency of LIMA1 knockdown were confirmed through Western blot analysis (Fig. 3B, C). In LTH-sEV^shCtr^ or LTH-sEV^shLIMA1^ treated LX2, we observed that both pKH26-labeled LTH-sEV^shCtr^ and LTH-sEV^shLIMA1^ were internalized by LX2 and distributed around the nucleus after co-culture for 6 h (Fig. 3D). Compared to LX2 without sEV treatment, LTH-sEV^shCtr^ promoted the expression of fibrosis-related markers COL1A1, COL3A1, and α-SMA in LX2. However, the expression of these markers was significantly down-regulated by LTH-sEV^shLIMA1^ (Fig. 3E–I). CCK8 results also revealed that LTH-sEV^shCtr^ accelerated the activation and proliferation of LX2, but this effect was weakened in LX2 treated with LTH-sEV^shLIMA1^ (Fig. 3J). Furthermore, we examined the direct regulatory effect of LIMA1 overexpression or knockdown on HSCs activation. In LX2 transfected with different doses of pCDNA-LIMA1, LIMA1 overexpression significantly increased the mRNA and protein expression of COL1A1, COL3A1, and α-SMA in LX2, as well as promoted their proliferation (Fig. 4A–F). On the other hand, LX2 transfected with LIMA1 siRNA (siLIMA1) showed reduced mRNA and protein expression of COL1A1, COL3A1, and α-SMA, as well as inhibited proliferation compared to the control group (transfected with Control siRNA (siCtr)) (Fig. 4G–L). The sEV concentration increased while the size distribution remained unchanged in L02^shLIMA1^ cells treated with OPA (Additional file 1: Figure S7A–C). These results indicate that LIMA1 has no impact on the formation of sEVs. These findings confirm the significant role of LIMA1 in LTH-sEV promoted LX2 activation and demonstrate its ability to regulate LX2 activation in vitro.

### LTH-sEV derived LIMA1 promotes HSCs activation through inhibiting mitophagy

**Fig. 5:**
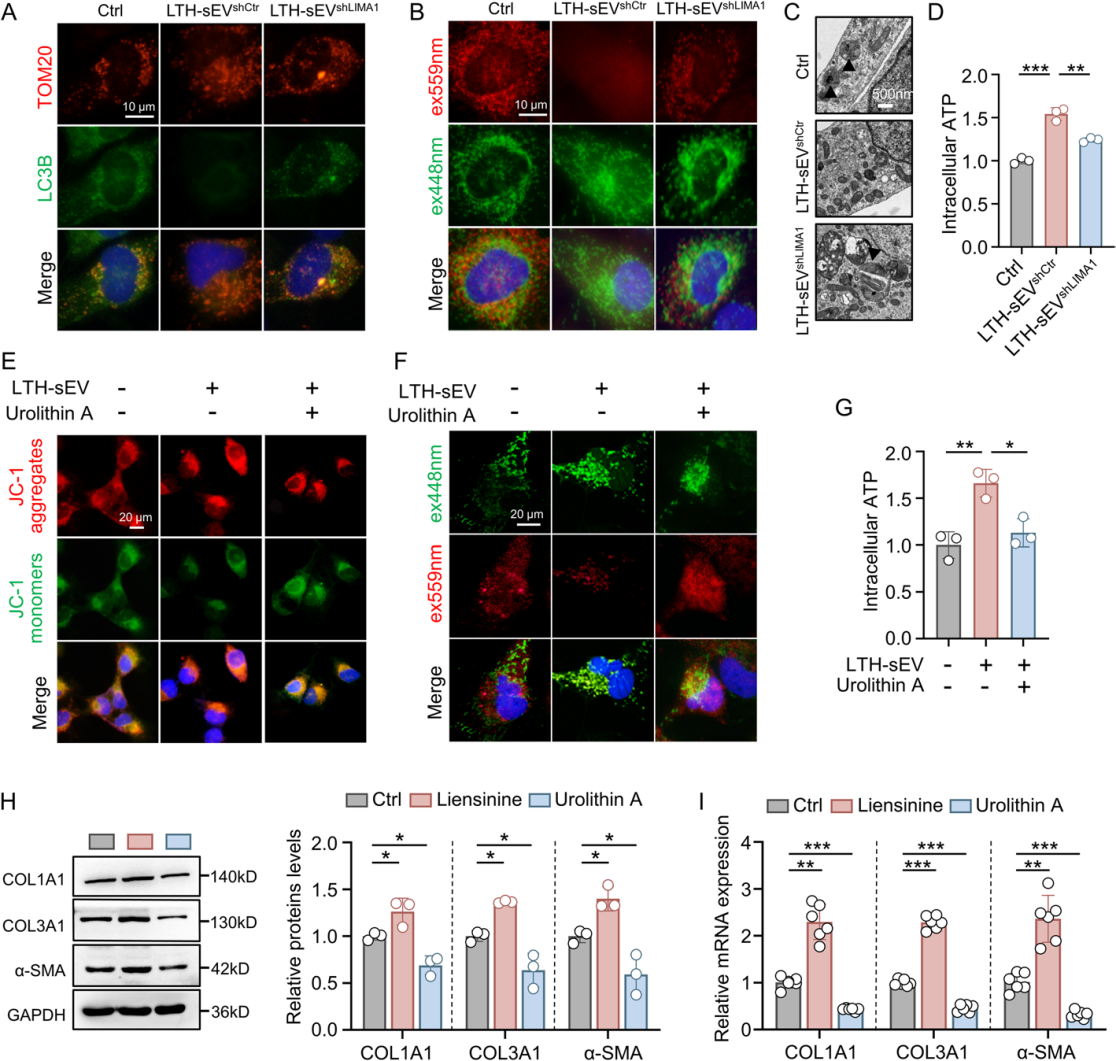
LTH-sEV derived LIMA1 inhibits mitophagy in LX2. **A** Immunofluorescence staining showing LC3B (green) and TOM20 (red) in LX2 treated with LTH-sEV^shCtr^ or LTH-sEV^shLIMA1^. Scale bar = 10 μm. **B** LTH-sEV^shCtr^ or LTH-sEV^shLIMA1^ treated LX2 were transfected with mitochondrially targeted mKeima and excitation at 550 nm (red) and 438 nm (green) by microscopy. Scale bar = 10 µm. **C** Autophagic microstructures in LX2 mitochondria were examined by transmission electron microscopy. Scale bars, 500 nm. Black arrowheads, mitophagy. **D** ATP content in LX2 treated with LTH-sEV^shCtr^ or LTH-sEV^shLIMA1^ were measured. **E** The mitochondrial membrane potential of LX2 was determined by JC-1 staining. Scale bar = 20 μm. **F** Different treated LX2 were transfected with mitochondrially targeted mKeima and excitation at 550 nm (red) and 438 nm (green) by microscopy. Scale bar = 20 µm. **G** ATP levels of LX2 were determined using an ATP determination kit. **H** Western blot of COL1A1, COL3A1 and α-SMA in LX2 treated with Liensinine or Urolithin A. **I** qRT-PCR of COL1A1, COL3A1 and α-SMA mRNA in LX2 treated with Liensinine or Urolithin A. All data were expressed as the means ± SD of at least 3 independent experiments, *P < 0.05; **P < 0.01; ***P < 0.001; ****P < 0.0001

We investigated the mechanism by which LTH-sEV transports LIMA1 to activate LX2. Previous studies have shown that inhibition of mitophagy disrupts mitochondrial homeostasis, activates HSCs and accelerates the progression of liver fibrosis [12]. Building on this knowledge, we hypothesized that LIMA1 might promote HSCs activation by inhibiting mitophagy. To test this hypothesis, we first examined the effect of LTH-sEV on mitophagosome formation in LX2. Our findings revealed a decrease in the co-localization of TOM20 and LC3, markers for mitochondria and autophagosomes respectively, in LX2 treated with LTH-sEV^shCtr^ and pLTH-sEV compared to the control group (Fig. 5A and Additional file 1: Fig. S8A). However, in LX2 treated with LTH-sEV^shLIMA1^, the co-localization was restored (Fig. 5A). These results suggest that LTH-sEV^shCtr^ can hinder the formation of mitophagosomes, while the number of mitophagosomes unchanged in the LTH-sEV^shLIMA1^ group. Additionally, we used the pH-sensitive fluorescent protein mt-keima-COX8 to assess mitochondrial lysosomes in LX2. Our observations indicated a decrease in mt-Keima fluorescence within lysosomes in the LTH-sEV^shCtr^ and pLTH-sEV group compared to the control group (Fig. 5B and Additional file 1: Fig. S8B). In contrast, the LTH-sEV^shLIMA1^ group showed an increase in mt-Keima fluorescence within lysosomes (Fig. 5B). Furthermore, electron microscopy analysis revealed a higher presence of mitophagosome-like structures in LX2 treated with LTH-sEV^shCtr^ compared to the LTH-sEV^shLIMA1^ group, which exhibited a reduction in the number of mitophagosome (Fig. 5C). Mitochondria play a crucial role in adenosine triphosphate (ATP) production and serve as pivotal organelles for cellular energy conversion. Therefore, we assessed ATP production in LX2 and found that the LTH-sEV^shLIMA1^ group exhibited lower ATP production compared to LTH-sEV^shCtr^ (Fig. 5D). To further verify the role of LTH-sEV in LX2 mitophagy, the mitophagy promoter Urolithin A (UA) was used to supplement it. It was found that after supplementing with UA, the LX2 mitochondrial membrane potential decreased (Fig. 5E) and the LTH-sEV-mediated mitochondrial inhibitory effect was restored (Fig. 5F), ATP production is reduced (Fig. 5G). At the same time, LX2 was treated with the mitophagy inhibitor Liensinine and the mitophagy promoter UA respectively. The results showed that Liensinine could increase the protein and mRNA expression of fibrosis-related markers α-SMA, COL1A1, and COL3A1. However, UA reduced the protein and mRNA expression of α-SMA, COL1A1, and COL3A1 (Fig. 5H-I). Thus, mitophagy play an important role in LTH-sEV mediated HSC activation in LX2.

**Fig. 6:**
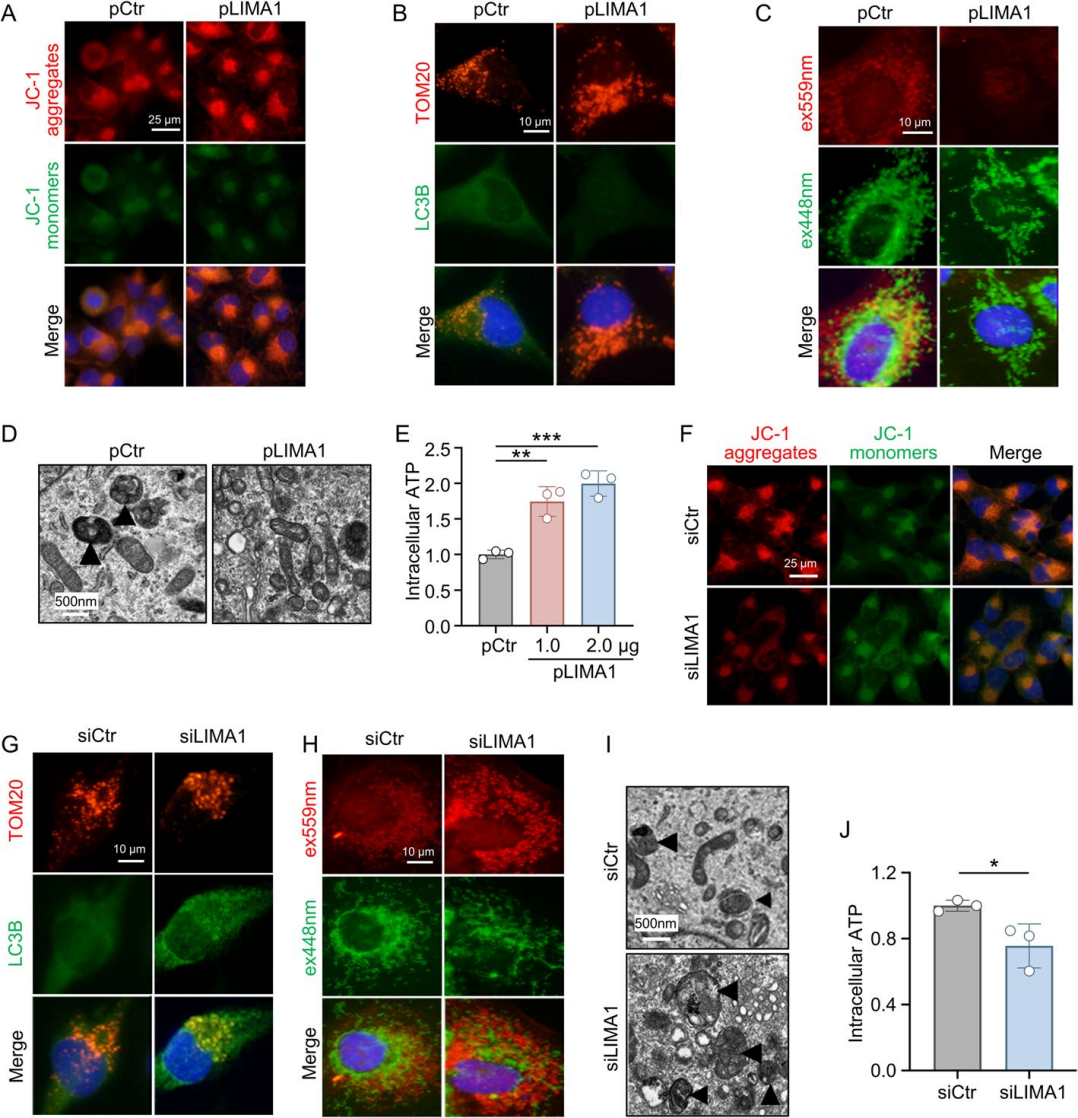
LIMA1 overexpression and knockdown promote or inhibit HSCs mitophagy. **A** Mitochondrial membrane potential of LX2 overexpressing LIMA1 was measured by JC-1 staining. Scale bar = 25 μm. **B** Immunofluorescence staining shows LC3B (green) and TOM20 (red) fluorescence of LX2 overexpressing LIMA1. Scale bar = 10 μm. **C** LX2 overexpressing LIMA1 was transfected with mitochondria-targeted mKeima and microscopically excited at 550 nm (red) and 438 nm (green). Scale bar = 10 µm. **D** Autophagic microstructures in LX2 overexpressing LIMA1 mitochondria were examined by transmission electron microscopy. Scale bars = 500 nm. Black arrowheads, mitophagy. **E** ATP levels of LX2 overexpressing LIMA1 were determined using an ATP determination kit. **F** The mitochondrial membrane potential of LIMA1-knockdown LX2 was determined by JC-1 staining. Scale bar = 25 μm. **G** Autophagic microstructures in LIMA1-knockdown LX2 mitochondria were examined by transmission electron microscopy. Scale bars = 500 nm. **H** Immunofluorescence staining showing LC3B (green) and TOM20 (red) in LIMA1-knockdown LX2. Scale bar = 10 μm. **I** LIMA1-knockdown LX2 were transfected with mitochondrially targeted mKeima and excitation at 550 nm (red) and 438 nm (green) by microscopy. Scale bar = 10 µm. **J** ATP levels of LIMA1-knockdown LX2 were determined using an ATP determination kit. All data were expressed as the means ± SD of at least 3 independent experiments, *P < 0.05; **P < 0.01; ***P < 0.001

The effects of overexpression and knockdown of LIMA1 on mitophagy of LX2 were also observed. JC-1 staining showed that the mitochondrial membrane potential of LX2 overexpressing LIMA1 was increased (Fig. 6A). In LX2 overexpressing LIMA1, there was a reduction in the colocalization of LC3B and TOM20 (Fig. 6B), a decrease in the number of lysosomal mt-Keima (Fig. 6C), an increase in the number of mitochondria (Fig. 6D), and an increase in the amount of ATP levels (Fig. 6E). In contrast, knocking down LIMA1 led to a reduction in mitochondrial membrane potential (Fig. 6F), an elevation in the co-localization of LC3B and TOM20 (Fig. 6G), an augmentation in the quantity of lysosomal mt-Keima (Fig. 6H), a decline in the number of mitochondria (Fig. 6I), and a decrease in ATP levels (Fig. 6J). These findings suggest that LTH-sEV-derived LIMA1 may promote LX2 activation by inhibiting mitophagy.

### LTH-sEV delivered LIMA1 inhibits PINK1 mediated mitophagy through by promoting PINK1 degradation

**Fig. 7:**
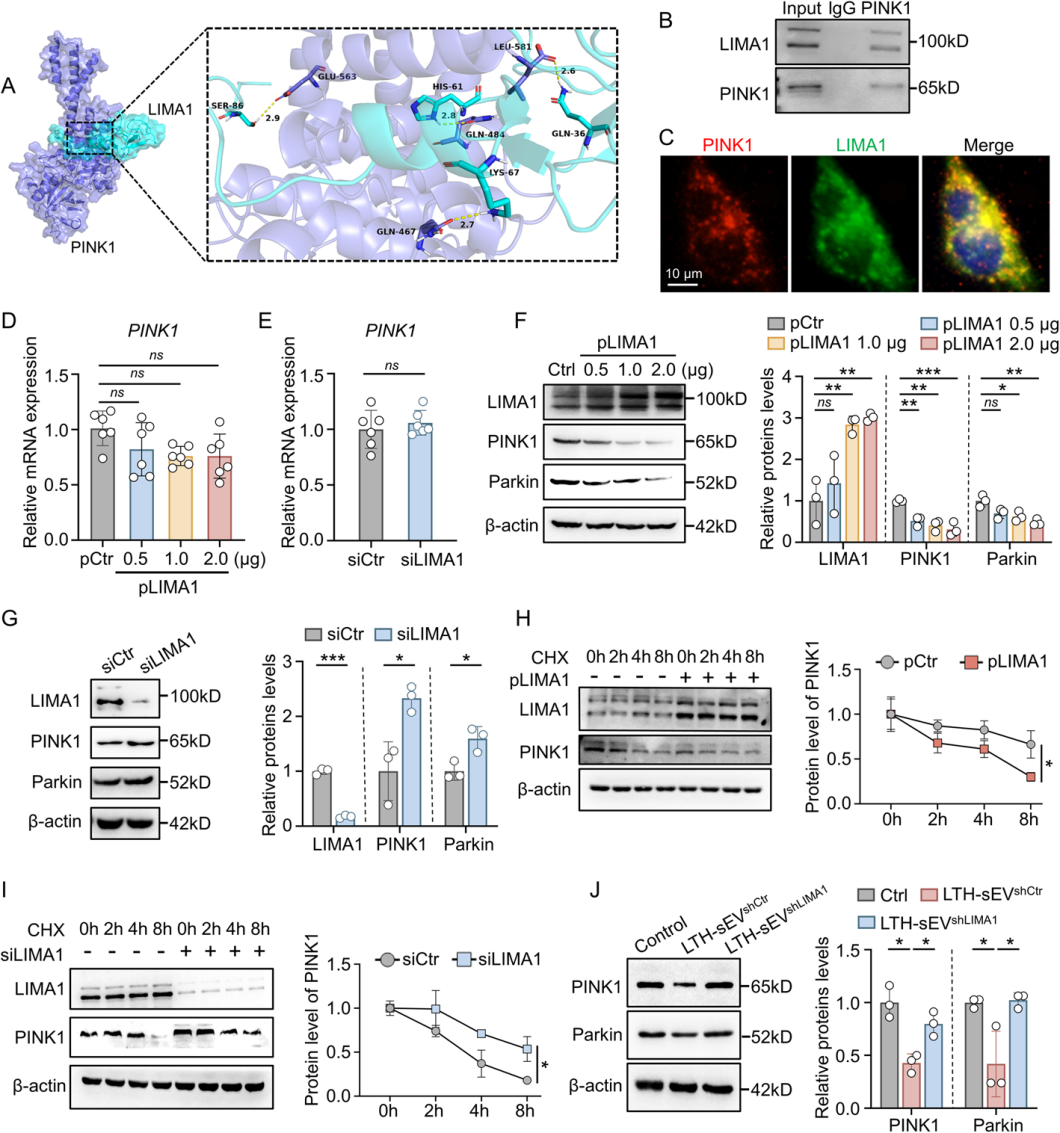
LIMA1 regulates mitochondrial autophagy and reduces its stability by interacting with PINK1. **A** Graphical representation of three-dimensional structures of the interaction model of LIMA1 with PINK1. **B** Co-IP analysis of the endogenous interaction of LIMA1 and PINK1 in LX2. **C** The co-localization between LIMA1 (green) with PINK1 (red) was analyzed by confocal microscopy in LX2. Scale bar = 10 μm. **D** qRT-PCR of PINK1 mRNA in LX2 with LIMA1 overexpression. Expression of PINK1 mRNA was quantified relative to β-actin. **E** qRT-PCR of PINK1 mRNA in LX2 with LIMA1 knockdown. **F** Western blot of PINK1 and Parkin in LIMA1-overexpressing plasmid-transfected LX2. **G** Western blot of PINK1 and Parkin in LIMA1-knockdown LX2. **H** LX2 overexpressing LIMA1 were treated with CHX to inhibit protein synthesis, and PINK1 protein turnover was analyzed over time. **I** Similarly, LX2 with knockdown of LIMA1 were also treated with CHX and to measure PINK1 protein levels. **J** Western blot of PINK1 and Parkin in LTH-sEV^shCtr^ or LTH-sEV^shLIMA1^ treated LX2. All data were expressed as the means ± SD of at least 3 independent experiments, *ns*: no significance; *P < 0.05; **P < 0.01; ***P < 0.001

The proteins that interact with LIMA1 and their structures were analyzed using the Protein–protein interaction (PPI) database hitpredict (http://www.hitpredict.org/) and PyMOL software. According to the molecular simulations, stable potential binding sites between LIMA1 and PINK1 docking were found (Fig. 7A). PINK1 is a crucial molecule involved in mitophagy. Based on this, we hypothesized that LIMA1 may regulate mitophagy by influencing PINK1. To investigate this further, we conducted immunoprecipitation (Co-IP) and fluorescence co-localization experiments (Fig. 7B, C), which confirmed the binding and co-localization of LIMA1 and PINK1 in LX2. We also examined the impact of LIMA1 on PINK1 expression and found that LIMA1 overexpression or knockdown did not significantly alter PINK1 mRNA levels (Fig. 7D, E), but overexpression of LIMA1 did reduce PINK1 protein expression (Fig. 7F). Conversely, knockdown of LIMA1 increased PINK1 protein expression (Fig. 7G). These findings suggest that LIMA1 may regulate PINK1 at the protein level rather than through gene transcription. To explore the time-dependent effect of LIMA1 on PINK1 protein degradation, we performed cycloheximide (CHX) pulse experiments. The results demonstrated that LX2 overexpressing LIMA1 had a significantly shorter half-life of PINK1 compared to control cells (Fig. 7H), whereas LX2 with LIMA1 knockdown showed a prolonged half-life of PINK1 protein (Fig. 7I). Additionally, we compared the effects of LTH-sEV^shCtr^ and LTH-sEV^shLIMA1^ on PINK1 expression in LX2. The results revealed that LTH-sEV down-regulated PINK1 protein, and this down-regulation was not observed after LIMA1 knockdown (Fig. 7J). In summary, our findings indicate that LTH-sEV-mediated transport of LIMA1 inhibits LX2 mitophagy, possibly by promoting the degradation of PINK1.

### LIMA1 knockdown inhibits the HSCs activation effect of LTH-sEV in vivo.

**Fig. 8:**
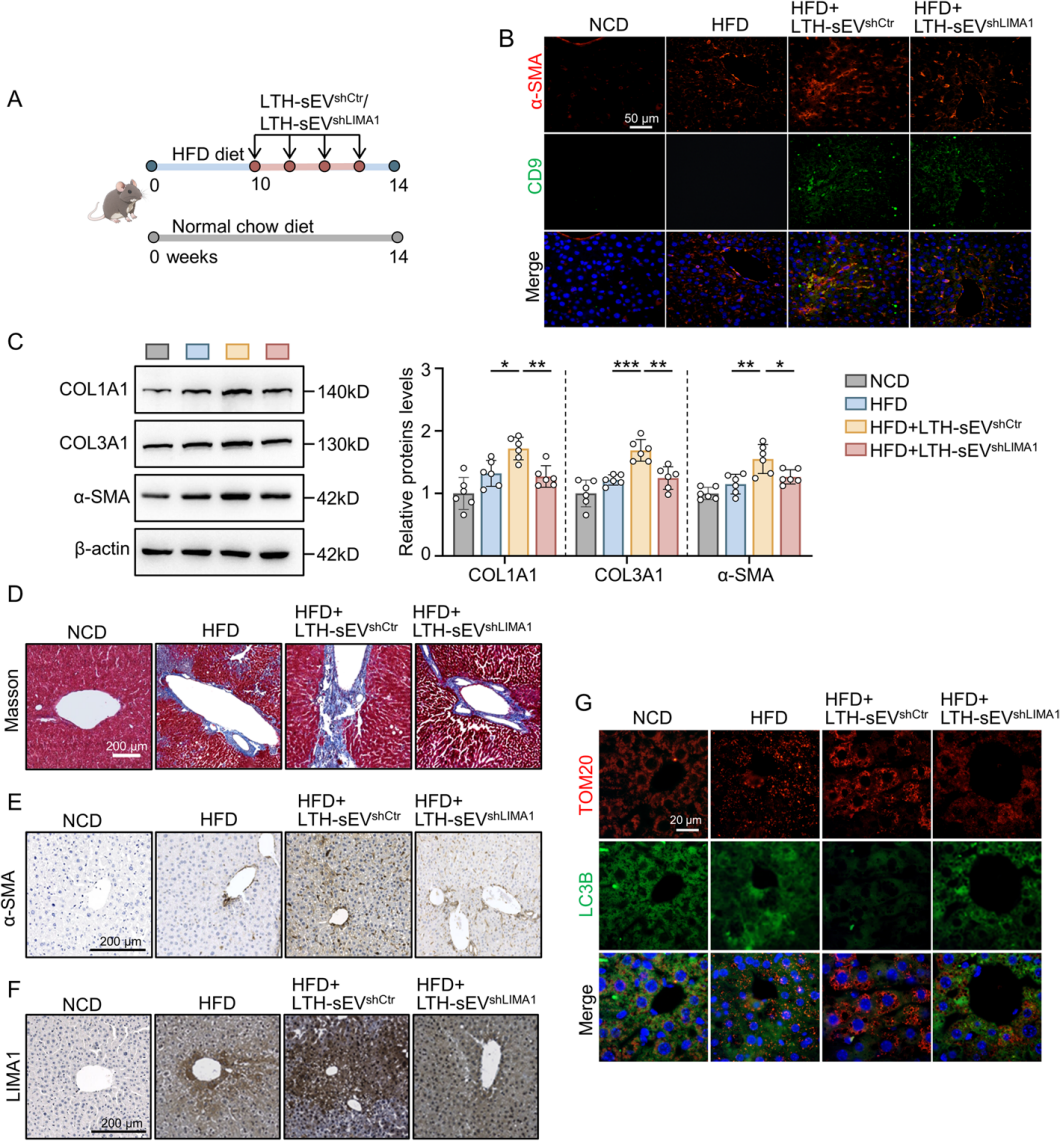
LTH-sEV derived LIMA1 transplantation worsens hepatic fibrosis in NAFLD mice. **A** C57BL/6 mice were placed normal chow diet or on high-fat diet and injected with LTH-sEV^shCtr^ or LTH-sEV^shLIMA1^ from the 10th week to the 14th week of HFD feeding. **B** Immunofluorescence staining of sEV marker CD9 (Red) and HSCs activation marker α-SMA (Green) in LTH-sEV^shCtr^ or LTH-sEV^shLIMA1^ treated mice livers. Scale bars = 50 μm, (*n* = 6 mice per group). **C** Western blot of COL1A1, COL3A1 and α-SMA in NCD group, HFD group, HFD + LTH-sEV^shCtr^ group and HFD + LTH-sEV^shLIMA1^ group (*n* = 6 mice per group). **D** Representative images of Masson staining in mice liver sections. Scale bars = 200 μm, (*n* = 6 mice per group). **E** Representative images of α-SMA immunohistochemical staining in mice liver sections. Scale bars = 200 μm, (*n* = 6 mice per group). **F** Representative images of LIMA1 immunohistochemical staining in mice liver sections. Scale bars = 200 μm, (*n* = 6 mice per group). **G** TOM20 (red IF) and LC3B (green IF) proteins were detected via IF staining. Scale bar = 20 μm, (*n* = 6 mice per group). All data were expressed as the means ± SD of at least 3 independent experiments, *P < 0.05; **P < 0.01

To investigate the role of LTH-sEV derived LIMA1 in promoting NAFLD-related liver fibrosis, we injected LTH-sEV^shCtr^ and LTH-sEV^shLIMA1^ into HFD mice through the tail vein (Fig. 8A). There was no significant difference in food intake of sEV-injected mice groups. (Additional file 1: Fig. S9). We performed double-label immunofluorescence using sEV marker CD9 and α-SMA, which revealed that LTH-sEV and LTH-sEV^shLIMA1^ were localized in α-SMA positive HSCs in the injected mice (Fig. 8B). Western blot analysis supported these findings, demonstrating that LTH-sEV^shCtr^ further enhanced the expression of liver fibrosis markers COL1A1, COL3A1, and α-SMA protein in the liver tissue of HFD mice. Protein detection in the LTH-sEV^shLIMA1^ group indicated that shLIMA1 knockdown reduced the promotion effect of LTH- sEV^shCtr^ on the expression of COL1A1, COL3A1, and α-SMA (Fig. 8C). Masson's trichrome staining, Sirius red staining and α-SMA immunohistochemistry revealed that LTH-sEV^shCtr^ significantly promoted collagen deposition and positive α-SMA expression in liver tissue in HFD mice. In contrast, the LTH-sEV^shLIMA1^ group exhibited reduced collagen deposition and decreased α-SMA expression (Fig. 8D, E and Additional file 1: Fig. S10). Similarly, the immunohistochemical results of LIMA1 showed the same trend (Fig. 8F). Mitophagy was significantly reduced in the HFD + LTH-sEV^shCtr^ group compared to the NCD group, HFD group, and HFD + LTH-sEV^shLIAM1^ group (Fig. 8G). These results suggest that LIMA1 knockdown can inhibit the effect of LTH-sEV on promoting HSCs activation in HFD mice. LIMA1 plays a crucial role in inhibiting autophagy and promoting HSCs activation and the progression of liver fibrosis by LTH-sEV.

## Discussion

Intercellular communication through sEV plays a crucial role in the progression of liver fibrosis in chronic liver diseases [19]. LIMA1-rich sEV is secreted by lipotoxic-damaged hepatocytes and activates HSCs, while LIMA1-knockdown sEV cannot activate HSCs. Therefore, we hypothesized that lipotoxic-damaged hepatocytes transport LIMA1 to HSCs via sEV, thereby promoting the expression of α-SMA and collagen in HSCs. When HSCs were treated with LTH-sEV or overexpressed LIMA1, the expression of PINK1 was down-regulated, resulting in the inhibition of mitophagy. Interestingly, inhibiting HSCs mitophagy using mitophagy inhibitor Liensinine promoted HSCs activation. Our findings demonstrate that in the HFD-induced liver injury model, sEV secreted by lipotoxically injured hepatocyte is enriched with LIMA1 and significantly contributes to the HSCs activation process.

Yamaguchi et al. showed that the tropism of sEV secreted by PA-treated hepatocytes toward HSCs increased. They speculated that integrin αVβ3 or α8β1 is highly expressed in activated HSCs. LTH-sEV may express high levels of integrins and their ligands, which readily bind to aHSCs [20]. Our study also showed that compared with LTH-sEV^shLIMA1^, LTH-sEV^shCtr^ have a higher tropism for HSCs. LIMA1, a cytoskeletal regulator containing LIM domains and multiple actin-binding domains [21], has been shown to regulate the cell movement and intercellular adhesion [22, 23]. In epithelial cell-derived tumors, the absence of LIMA1 can enhance tumor proliferation, invasion, migration, and angiogenesis [24, 25]. Additionally, it acts as a pivotal protein in regulating intestinal cholesterol absorption. However, the specific contribution of LIMA1 in HSCs activation and the progression of liver fibrosis remains unclear. This study discovered that sEV, abundant in LIMA1, is transported from lipotoxically damaged hepatocyte, leading to the promotion of HSCs activation both in vivo and in vitro. Knockdown of LIMA1 diminishes the HSCs activation function of sEV.

Mitophagy is a mitochondrial quality control mechanism that facilitates the targeted elimination of damaged mitochondria from the cell through the autophagy pathway. This process is closely associated with the development of chronic diseases. Inhibited mitophagy leads to lung fibroblast proliferation by activating the platelet-derived growth factor receptor/PI3K signaling pathway, resulting in pulmonary fibrosis [26]. AMPK agonists alleviate tubulointerstitial fibrosis by activating mitophagy [27]. However, the role of mitophagy in regulating HSCs activation remains uncertain. Previous studies have reported that mitophagy is inhibited during the activation of HSCs [28–30], and the activation of mitophagy induces HSCs apoptosis [12]. Conversely, some studies have found that mitophagy can promote HSCs activation [31, 32]. In this study, we treated HSCs with the mitophagy promoter Urolithin A and the mitophagy inhibitor Liensinine. Our findings revealed that the mitophagy promoter attenuated HSCs activation, while the mitophagy inhibitor promoted HSCs activation. These results confirm the role of mitophagy inhibition in HSCs activation and suggest that inhibiting mitophagy may promote the progression of liver fibrosis. The activation of HSCs is an energy-intensive process that necessitates a significant amount of intracellular ATP for synthesizing the extracellular matrix (ECM) [33]. Mitochondria are the primary site where cells utilize oxidative phosphorylation to generate ATP. We observed that LIMA1 and LIMA1 overexpression, carried by sEV in lipotoxic hepatocyte, can inhibit mitophagy induced by lipid deposition, increase the number of mitochondria, and promote ATP release. Therefore, we speculate that LIMA1-inhibited mitophagy may promote HSCs activation by enhancing ATP release.

PINK1 is a well-established protein involved in the regulation of the mitophagy signaling pathway. When mitochondria are damaged, PINK1 accumulates in the outer membrane of mitochondria through translocase of the outer membrane (TOM). It then activates and recruits Parkin, which in turn polyubiquitinates various mitochondrial protein substrates. In the presence of LC3 protein, PINK1 targets autophagosomes to mitochondria, leading to mitophagy [34]. In this study, we used AutoDock software to predict the proteins that bind to LIMA1, and found that LIMA1 can bind to the PINK1 protein. Although LIMA1 does not affect the expression of PINK1 mRNA, it does reduce the stability of PINK1 protein in LIMA1-overexpressing HSCs. Knockdown of LIMA1 sEV also diminishes the regulatory function of PINK1. These findings suggest that LIMA1 can regulate PINK1 protein at the post-translational level.

However, it is important to acknowledge the limitations of this study. Firstly, we only explored the role of LIMA1 in lipotoxic hepatocyte sEV-promoted HSCs activation at the animal model and cellular level. The expression levels of LIMA1 in liver tissue and serum sEV of clinical NAFLD patients are still unknown, and further investigation is needed to understand the relationship between LIMA1 and the progression of clinical liver fibrosis. Secondly, although we determined through software prediction and Co-IP experiments that LIMA1 can bind to PINK1 to regulate its stability, the specific binding site and mechanism of LIMA1 in regulating PINK1 stability remain unclear. Lastly, we observed that lipotoxic injury induced by OPA can promote sEV secretion in hepatocyte in a time-dependent manner, but the regulatory mechanism of sEV release in lipotoxic hepatocyte requires further study.

## Conclusions

LIMA1 can inhibit PINK1-Parkin-mediated mitophagy, thereby promoting HSC activation. LIMA1 enriched LTH-sEV play an important role in promoting HSC activation in NAFLD-associated liver fibrosis. These results provide new insights into the pathological mechanisms of fibrosis development in NAFLD.

### Supplementary Information

**Additional file 1: Fig. S1.** LIMA1 mRNA expression in MASH transcriptome data (GSE135251). **Fig. S2.** Cell types overexpressing LIMA1 in the liver of mice fed HFD. **Fig. S3.** Effects of OPA treatment on hepatocyte death. **Fig. S4.** Effects of pLTH-sEV on LX2 activation. **Fig. S5.** Effects of OPA treatment on LIMA1 content. **Fig. S6.** Effect of OPA treatment on LX2-derived sEV concentration and size distribution. **Fig. S7.** Effect of OPA treatment on L02-shLIMA1-derived sEV concentration and size distribution. **Fig. S8.** Effects of pLTH-sEV on LX2 mitophagy. **Fig. S9.** Food intake of sEV-injected mice groups. **Fig. S10.** Representative images of Sirus Red staining in mice liver sections.

**Additional file 2: Table S1.** Summary of primary antibodies used in Western Blot, Immunochemistry, immunofluorescence and Co-immunoprecipitation. **Table S2.** Primers used in this study. **Table S3.** RNA oligo used in this study.

## Data Availability

All data presented in the study are included in the manuscript and supplementary information file. Further inquiries regarding the datasets can be directed to the corresponding author.

## Abbreviations

ATP: Adenosine triphosphate
CCK8: Cell counting kit-8
CHX: Cyclohexane
COL1A1: Collagen type 1 alpha 1 chain
COL3A1: Collagen type 3 alpha 1 chain
Co-ip: Co-inmunoprecipitation
FDA: Food and Drug Administration
HSCs: Hepatic stellate cells
JC-1: 5,5ʹ,6,6ʹ-Tetrachloro-1,1ʹ,3,3ʹ-tetraethylbenzimidazolylcarbocyanine, iodide
LC3: Microtubule-associated protein 1 light chain-3
LIMA1: LIM domain and actin binding 1
L02-sEV: L02 derived small extracellular vesicles
LTH-sEV: Lipotoxic hepatocyte derived small extracellular vesicles
NAFLD: Nonalcoholic fatty liver disease
OPA: Oleic acid and palmitic acid
PBS: Phosphate buffered saline
pLIMA1: LIMA1 overexpression plasmid
PINK1: PTEN-induced kinase 1
qRT-PCR: Quantitative real-time polymerase chain reaction
SEM: Standard error of mean
α-SMA: Alpha smooth muscle actin
siLIMA1: LIMA1 small interfering RNA
TEM: Transmission electron microscope
TOM20: Translocase of outer mitochondrial membrane 20
