# Supplementary material for: Lipotoxic hepatocyte derived LIMA1 enriched small extracellular vesicles promote hepatic stellate cells activation via inhibiting mitophagy
Source: Cell Mol Biol Lett. 2024 May 31;29:82. doi: 10.1186/s11658-024-00596-4 (PMC11140962; doi:10.1186/s11658-024-00596-4)
Supplement: Supplementary file 1 — Additional file 1: Fig. S1. LIMA1 mRNA expression in MASH transcriptome data (GSE135251). Fig. S2. Cell types overexpressing LIMA1 in the liver of mice fed HFD. Fig. S3. Effects of OPA treatment on hepatocyte death. Fig. S4. Effects of pLTH-sEV on LX2 activation. Fig. S5. Effects of OPA treatment on LIMA1 content. Fig. S6. Effect of OPA treatment on LX2-derived sEV concentration and size distribution. Fig. S7. Effect of OPA treatment on L02-shLIMA1-derived sEV concentration and size distribution. Fig. S8. Effects of pLTH-sEV on LX2 mitophagy. Fig. S9. Food intake of sEV-injected mice groups. Fig. S10. Representative images of Sirus Red staining in mice liver sections. [file 11658_2024_596_MOESM1_ESM.docx]

**Additional file 1**


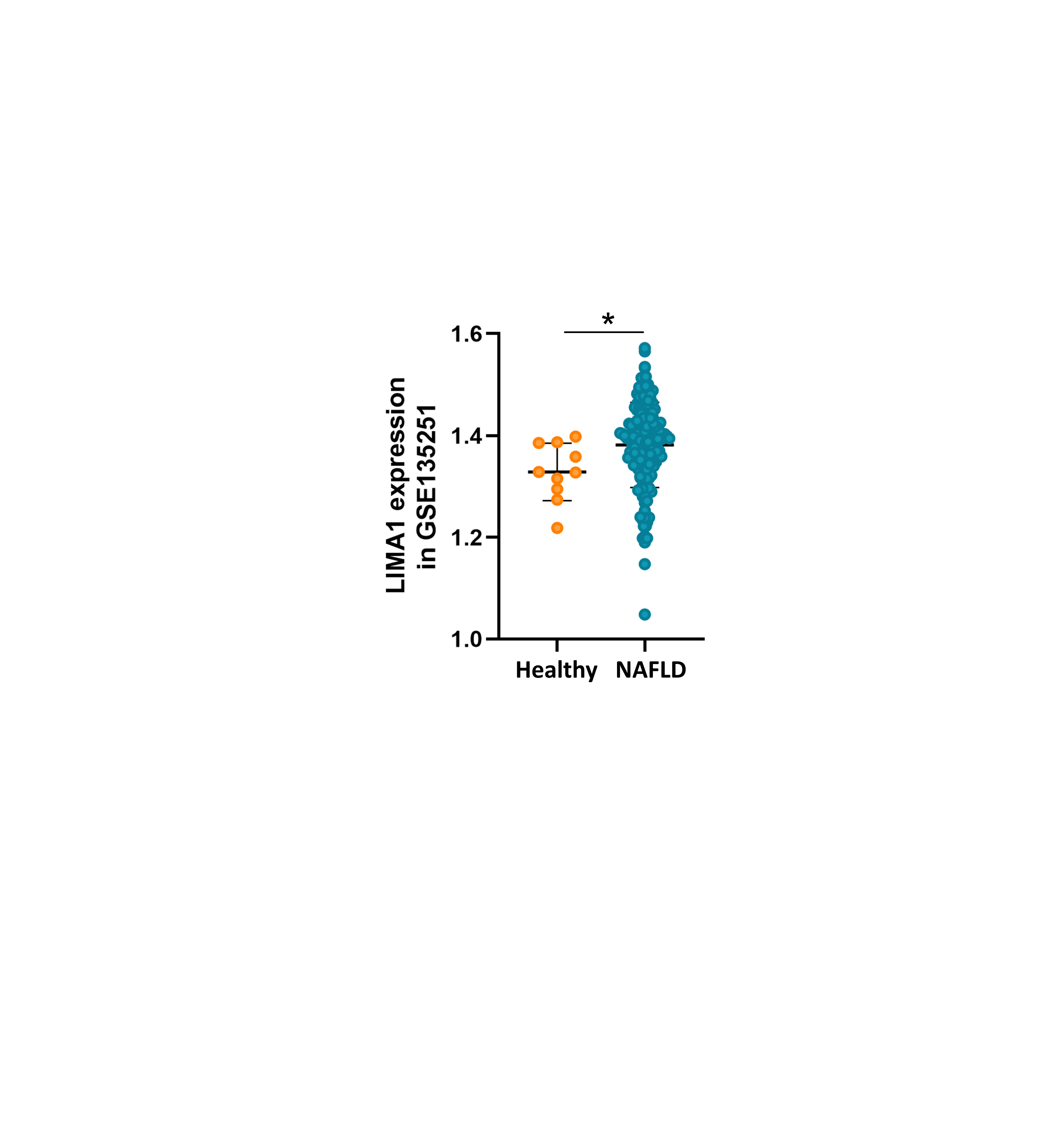


**Fig. S1. LIMA1 mRNA expression in MASH transcriptome data (GSE135251). * *P* < 0.05.**


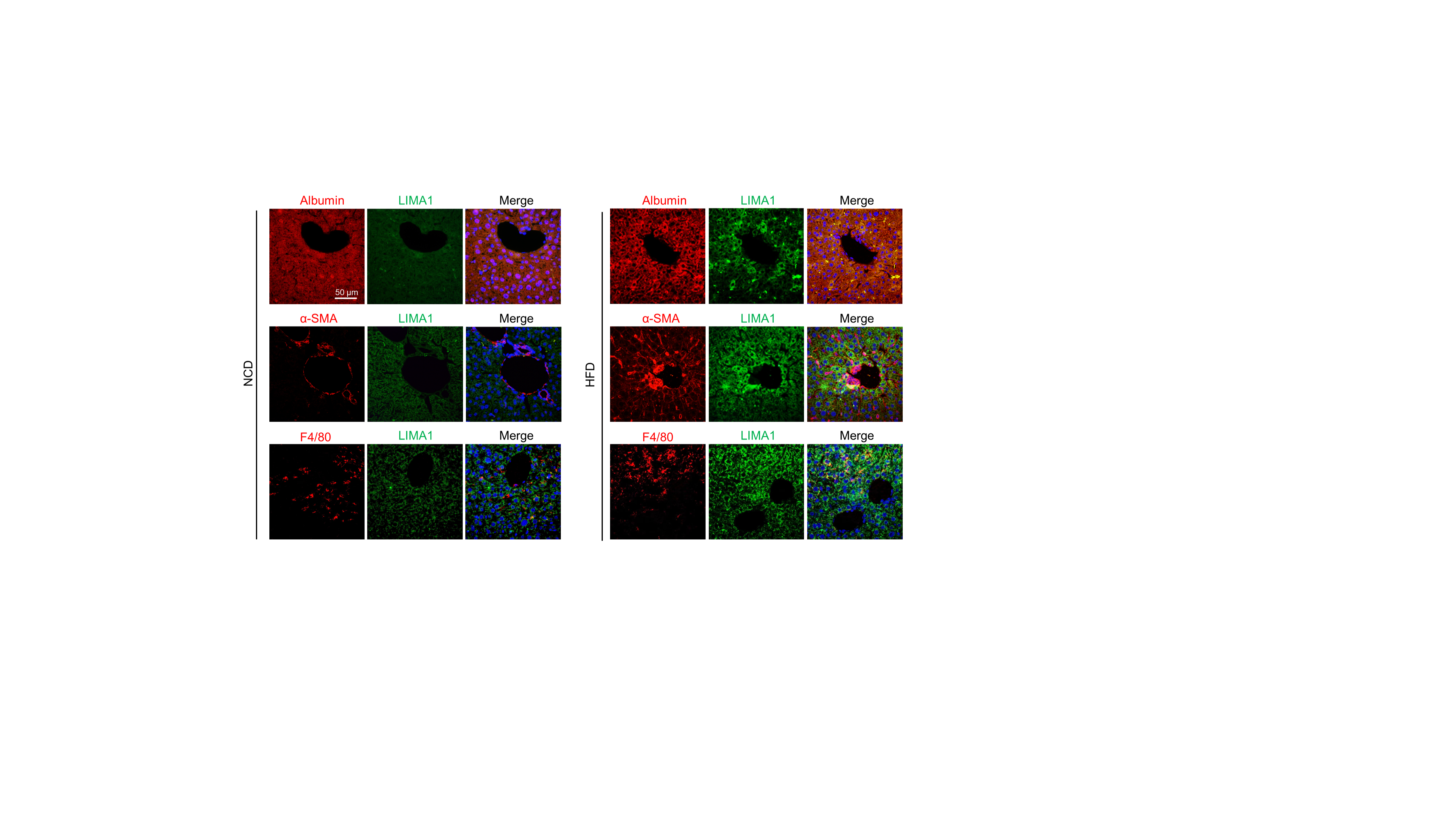


**Fig. S2. Cell types overexpressing LIMA1 in the liver of mice fed HFD.**

Immunofluorescence stain of LIMA1 and Albumin, LIMA1 and α-SMA, LIMA1 and F4/80 in liver tissues of NCD and HFD mice. Scale bar = 50 μm, (n = 6 mice per group).


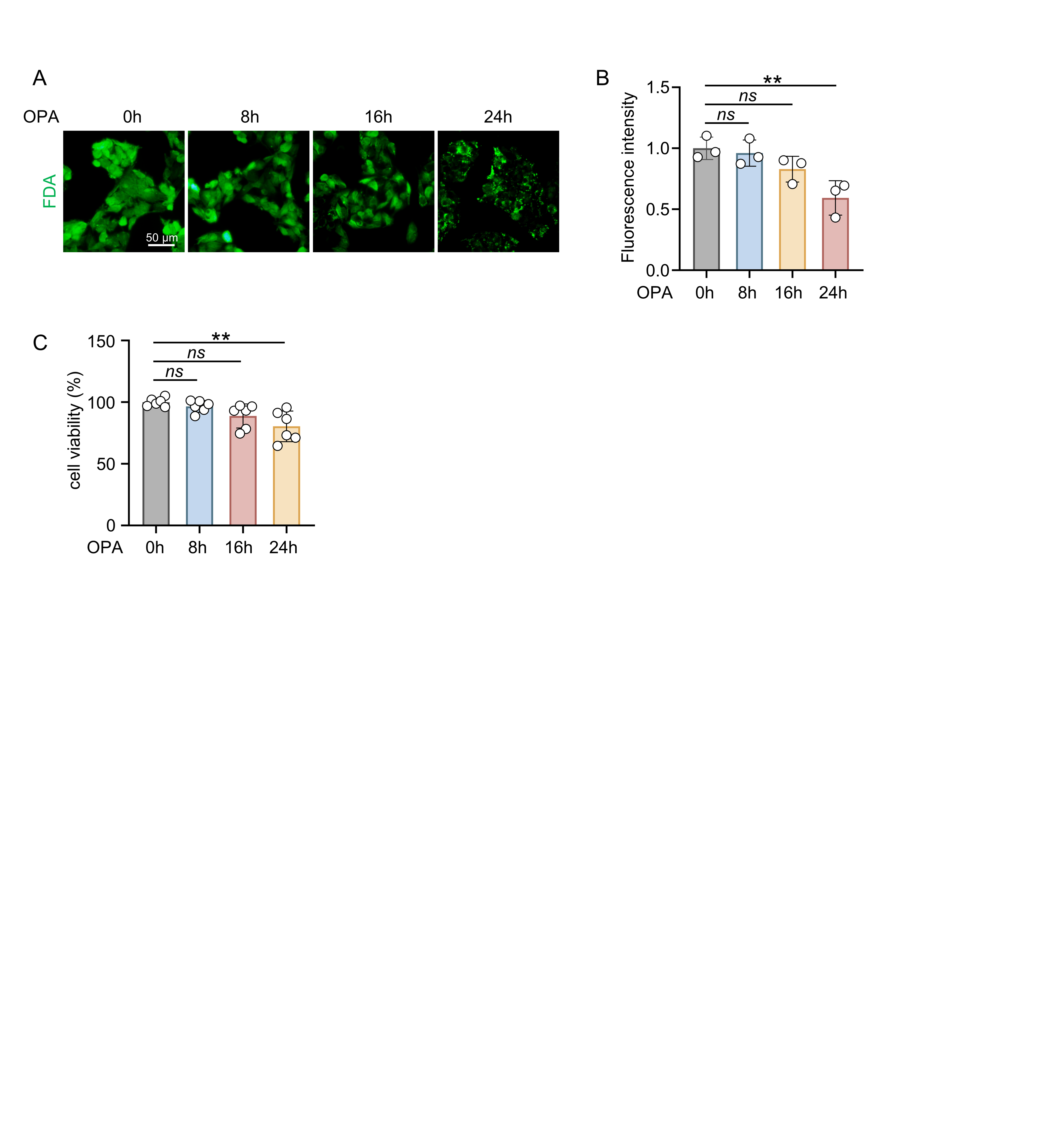


**Fig. S3. Effects of OPA treatment on hepatocytes death.**

**A** Representative images of L02 treated with OPA at different times visualized by fluorescein diacetate (FDA) fluorescent staining. Scale bars = 50 μm. **B** The FDA fluorescence intensity represents the mean of 3 independent images. **C** Cell viability detection of OPA treated LX2 were determined by CCK-8 assays. All data were expressed as the means ± SD of at least 3 independent experiments, ns: no significance, ** *P* < 0.01.


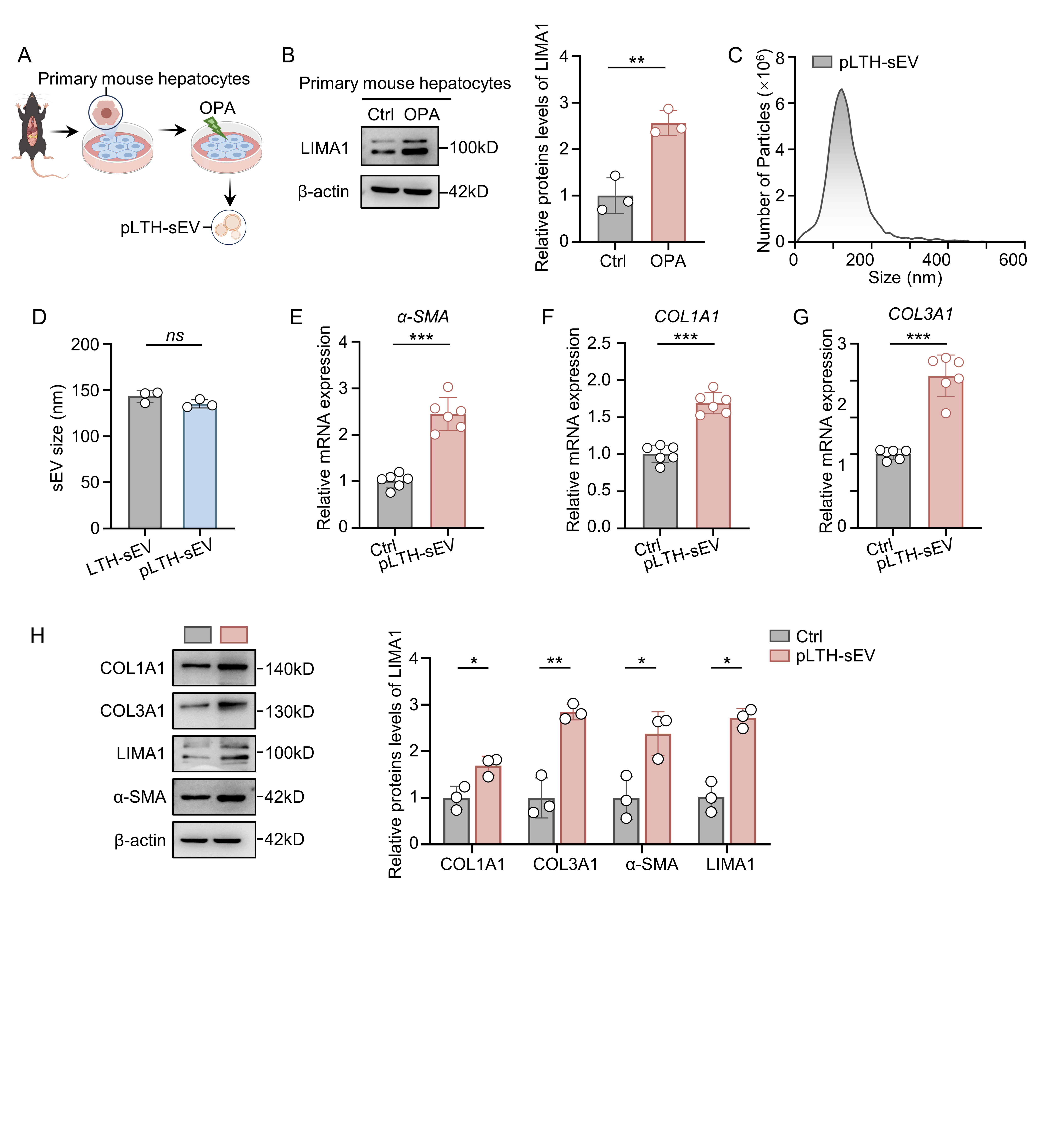


**Fig. S4. Effects of pLTH-sEV on LX2 activation.**

**A** Schematic diagram of primary mouse hepatocyte processing. **B** Western blot of LIMA1 in primary mouse hepatocyte treated with OPA. **C, D** Representative graph of pLTH-sEV concentration and size distribution as measured by nanoparticle tracking analysis. **E-G** qRT-PCR of COL1A1, COL3A1 and α-SMA mRNA in LX2 treated with pLTH-sEV. **H** Western blot of COL1A1, COL3A1, α-SMA and LIMA1 in LX2 treated with pLTH-sEV. All data were expressed as the means ± SD of at least 3 independent experiments, ns: no significance, * *P* < 0.05; ** *P* < 0.01; *** *P* < 0.001.


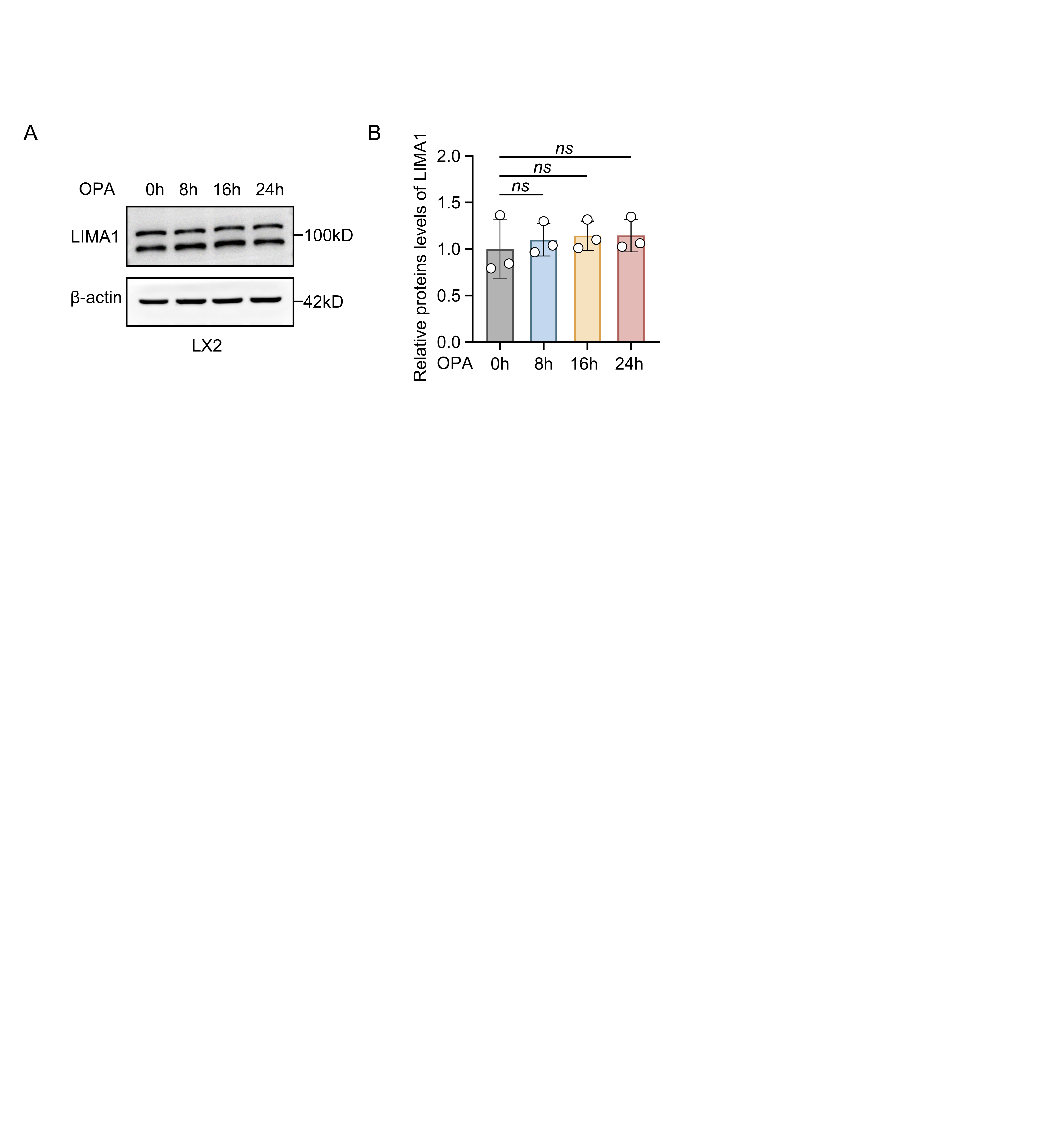


**Fig. S5.** **Effects of OPA treatment on LIMA1 content in LX2 cells.**

**A** Western blot of LIMA1 expression level of OPA treated LX2 at different times. B Quantification of LIMA1 protein level relative to β-actin protein. All data were expressed as the means ± SD of 3 independent experiments, ns: no significance.


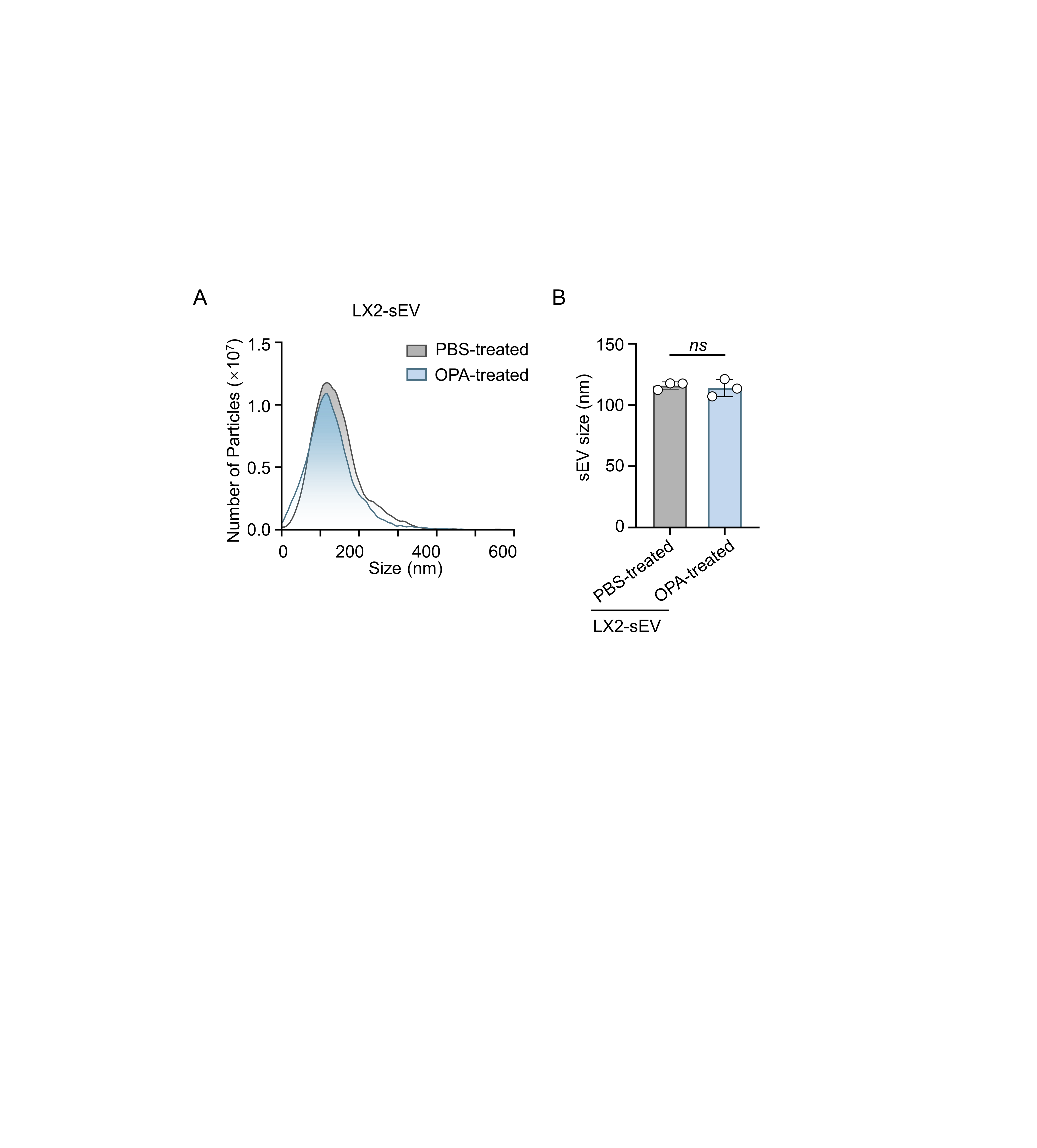


**Fig. S6.** **Effect of OPA treatment on LX2-derived sEV concentration and size distribution.**

**A** Impact of OPA treatment on LX2-derived sEV concentration was detected by nanoparticle tracking analysis. **B** Effect of OPA treatment on LX2-derived sEV size distribution. All data were expressed as the means ± SD of 3 independent experiments, ns: no significance.


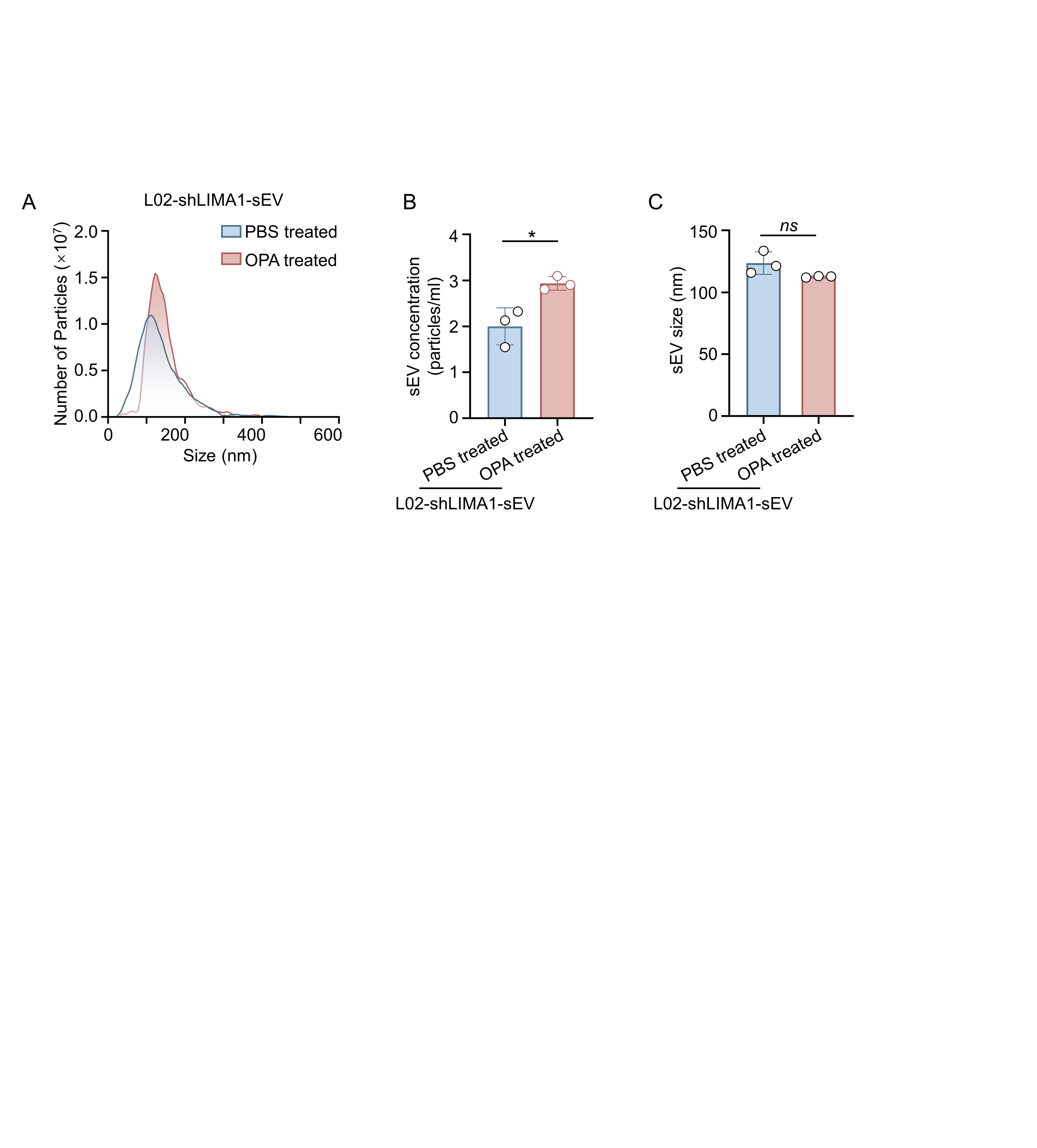


**Fig. S7.** **Effect of OPA treatment on L02-shLIMA1-derived sEV concentration and size distribution.**

**A** The size and concentration of LTH-sEV were determined by nanoparticle tracking analysis. **B** The concentration of LTH-sEV after OPA treatment of L02 for different times. **C** Size distribution of LTH-sEV after OPA treatment of L02 for different times. All data were expressed as the means ± SD of 3 independent experiments, ns: no significance, * *P* < 0.05.


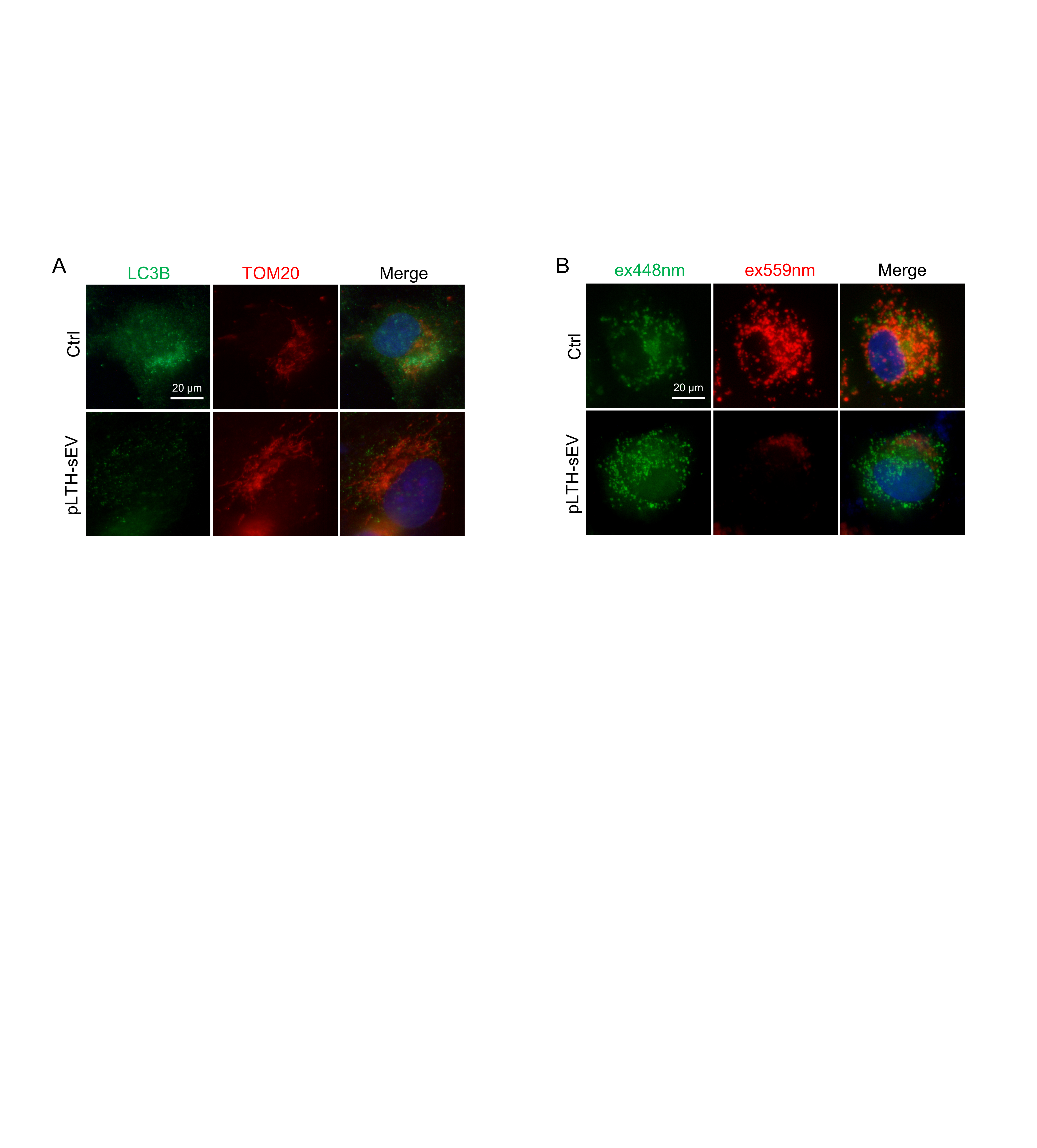


**Fig. S8.** **Effects of pLTH-sEV on LX2 mitophagy.**

**A** Immunofluorescence staining showing LC3B (green) and TOM20 (red) in LX2 treated with pLTH-sEV. Scale bar = 20 μm. **B** pLTH-sEV treated LX2 were transfected with mitochondrially targeted mKeima and excitation at 550 nm (red) and 438 nm (green) by microscopy. Scale bar = 20 µm.


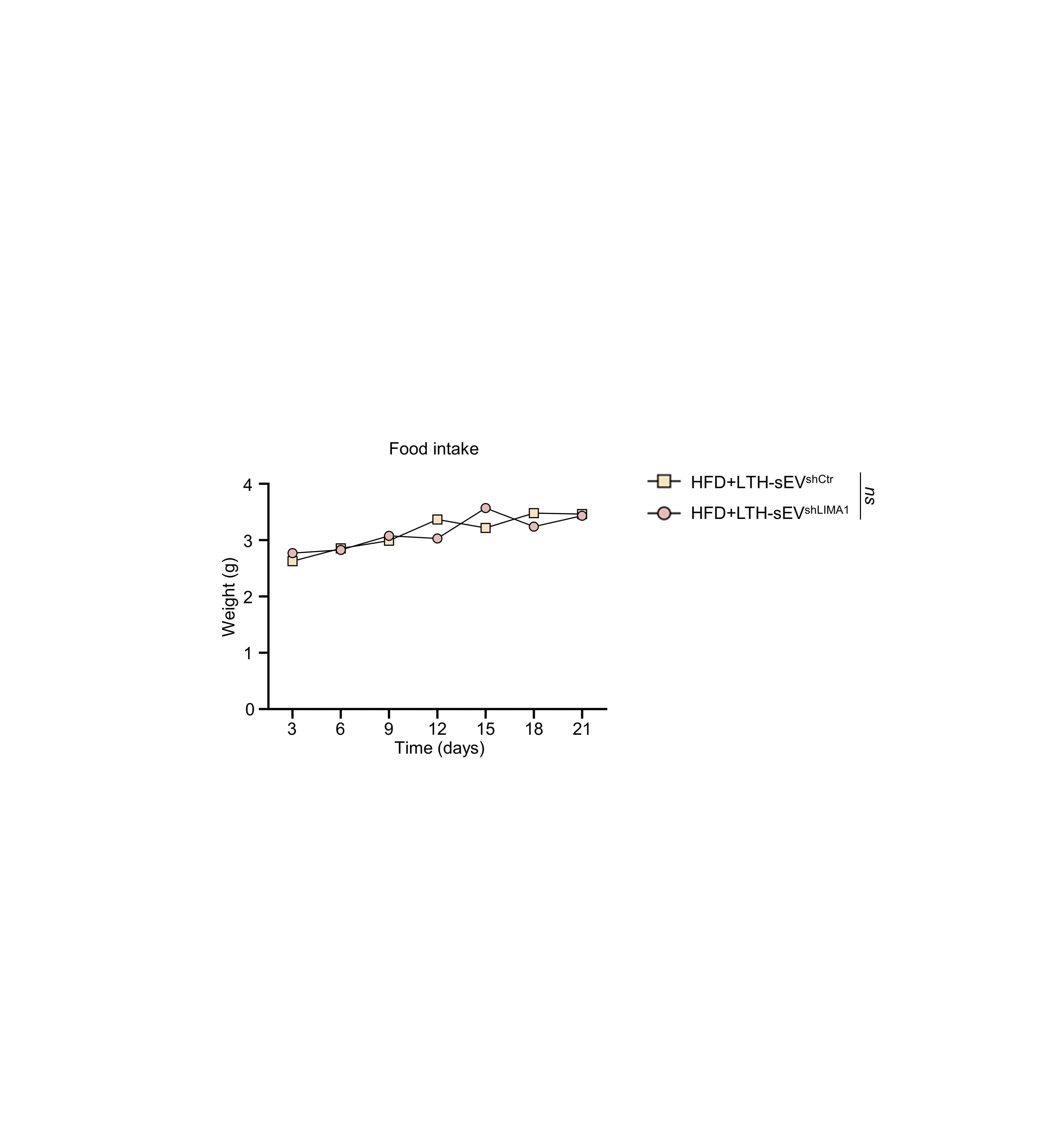


**Fig. S9.** **Food intake of sEV-injected mice groups.**

Food intake per mice per day. Food intake per cage was measured daily during 21 days and divided by the number of mice per cage. Each cage contains 6 mice. ns: no significance.


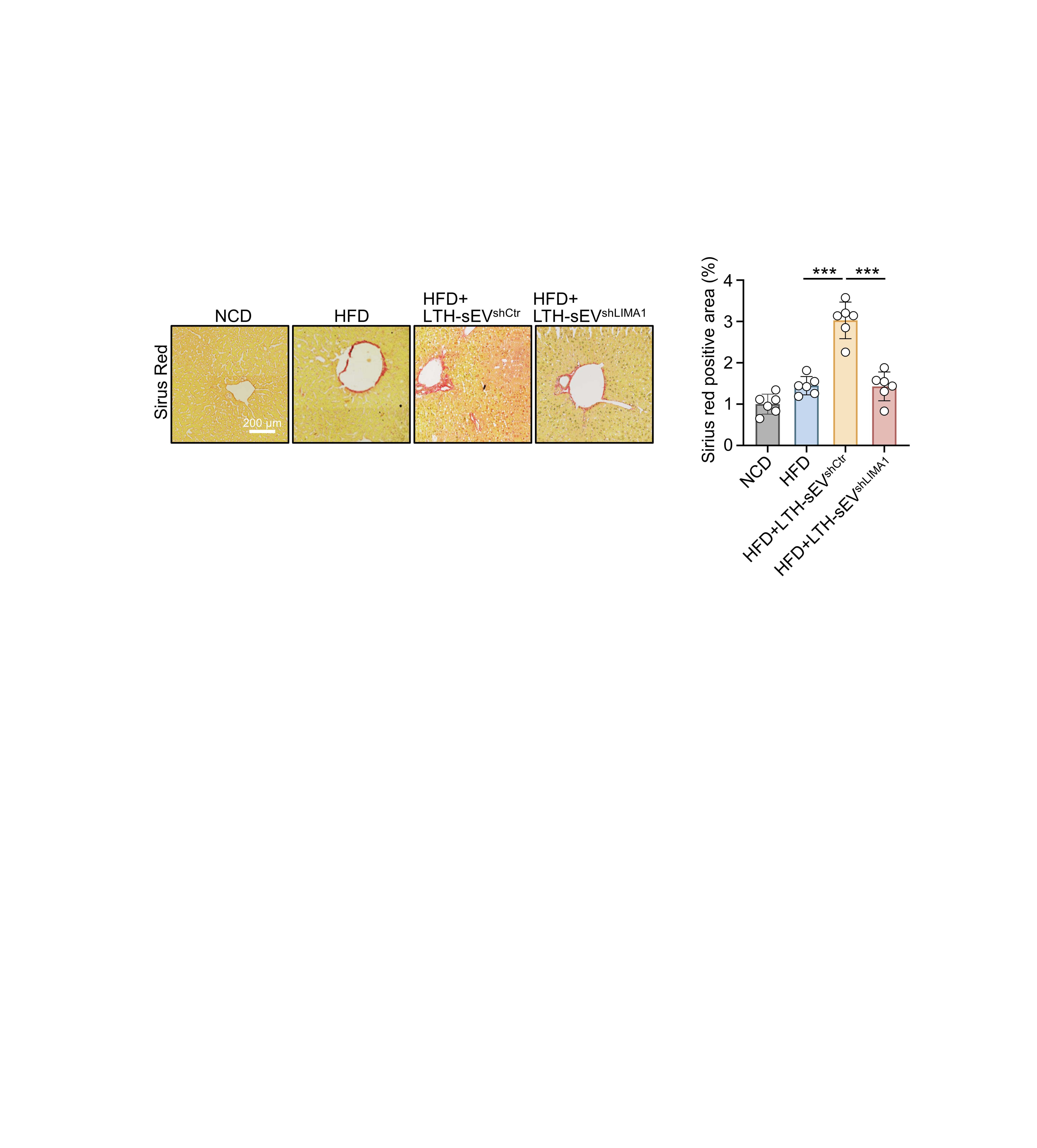


**Fig. S10. Representative images of Sirus Red staining in mice liver sections.**

Representative images of Sirus Red staining in mice liver sections. Scale bars = 200 μm, Each group contained six mice, with each point representing one mice. *** *P* < 0.001.
