# Supplementary material for: Lipotoxic hepatocyte derived LIMA1 enriched small extracellular vesicles promote hepatic stellate cells activation via inhibiting mitophagy
Source: Cell Mol Biol Lett. 2024 May 31;29:82. doi: 10.1186/s11658-024-00596-4 (PMC11140962; doi:10.1186/s11658-024-00596-4)
Supplement: Supplementary file 2 — Additional file 2: Table S1. Summary of primary antibodies used in Western Blot, Immunochemistry, immunofluorescence and Co-immunoprecipitation. Table S2. Primers used in this study. Table S3. RNA oligo used in this study. [file 11658_2024_596_MOESM2_ESM.docx]

**Additional file 2**

**Supplementary Table 1. Summary of primary antibodies used in Western Blot, Immunochemistry, immunofluorescence and Co-immunoprecipitation.**

| Concentration | | | | | | |
| --- | --- | --- | --- | --- | --- | --- |
| Antibody | for WB | for IHC | for IF | for co-IP | Cat. No. | Company |
| LIMA1 | 1:1000 | 1:100 | 1:100 | 1:50 | 66071-1-lg | Proteintech |
| COL1A1 | 1:1000 | / | / | / | A1352 | Abclonal |
| COL3A1 | 1:1000 | / | / | / | A0817 | Abclonal |
| α-SMA | 1:1000 | 1:100 | 1:100 | / | A17910 | Abclonal |
| CD63 | 1:500 | / | / | / | A5271 | Abclonal |
| CD9 | 1:500 | / | 1:100 | / | A19027 | Abclonal |
| Calnexin | 1:500 | / | / | / | 10427-2-AP | Proteintech |
| TSG101 | 1:500 | / | / | / | A5789 | Abclonal |
| PINK1 | 1:1000 | / | / | 1:50 | A7131 | Abclonal |
| Parkin | 1:1000 | / | / | / | A0968 | Abclonal |
| β-actin | 1:5000 | / | / | / | AC006 | Abclonal |
| TOM20 | / | / | 1:100 | / | 11802-1-AP | Proteintech |
| LC3B | / | / | 1:100 | / | A17424 | Abclonal |
| GAPDH | 1:5000 | / | / | / | AC002 | Abclonal |
| F4/80 | / | / | 1:100 | / | 29414-1-AP | Proteintech |
| Albumin | / | / | 1:100 | / | 16475-1-AP | Proteintech |
| HRP Goat Anti-Rabbit IgG (H+L) | 1:5000 | / | / | / | AS014 | Abclonal |
| HRP Goat Anti-Mouse IgG (H+L) | 1:5000 | / | / | / | AS003 | Abclonal |
| Mouse Anti-Rabbit IgG LCS | / | / | / | 1:2000 | A25022 | IPKine |
| Goat Anti-Rabbit IgG HCS | / | / | / | 1:2000 | A25222 | IPKine |
| Cy3-Goat Anti-Mouse IgG (H+L) | / | 1:200 | / | / | AS008 | Abclonal |
| FITC-Goat Anti-Rabbit IgG (H+L) | / | 1:200 | / | / | AS011 | Abclonal |
| Cy3 Goat Anti-Rabbit IgG (H+L) | / | 1:200 | / | / | AS007 | Abclonal |
| FITC Goat Anti-Mouse IgG (H+L) | / | 1:200 | / | / | AS001 | Abclonal |

Abbreviation: Co-IP, immunoprecipitation; WB, western blot; IHC, immunohistochemistry; IF, immunofluorescence.

**Supplementary Table 2. Primers in this study.**

| Identifier | Forward (5’-3’) | Reverse (5’-3’) |
| --- | --- | --- |
| **For qPCR** | | |
| COL1A1 | TGGCAAAGACGGCAAAGG | AGGAGCACCAGCAGGACCATC |
| COL3A1 | CTCAGGGTGTCAAGGGTGAAA GTG | TGT ACC AGC CAG ACC AGG AAG AC |
| α-SMA | GTCATGGTCGGTATGGGTCAGAAAG | GTTGTAGAAGGTGTGGTGGTGCCAGATC |
| LIMA1 | CCAAGCCACTAAGTCCAGATTCCAG | GACATTCCACGCAGGTCTCTCTTG |
| PINK1 | GAAGCCAUCUUGAACACAATT | UUGUGUUCAAGAUGGCUUCTT |
| β-actin | GGCCAACCGCGAGAAGATGAC | GGATAGCACAGCCTGGATAGCAAC |

Abbreviation: qPCR, quantitative real-time polymerase chain reaction.

**Supplementary Table 3. RNA oligo in this study.**

| Identifier | Sense | Antisense |
| --- | --- | --- |
| **For Knockdown** | | |
| LIMA1 oligo | GCCCAAAGCCGAAGUGCAATT | UUGCACUUCGGCUUUGGGCTT |
| Control oligo | UUCUCCGAACGUGUCACGUTT | ACGUGACACGUUCGGAGAATT |
